# Crystal structures of four co-crystals of (*E*)-1,2-di(pyridin-4-yl)ethene with 4-alk­oxy­benzoic acids: 4-meth­oxy­benzoic acid–(*E*)-1,2-di(pyridin-4-yl)ethene (2/1), 4-eth­oxy­benzoic acid–(*E*)-1,2-di(pyridin-4-yl)ethene (2/1), 4-*n*-propoxybenzoic acid–(*E*)-1,2-di(pyridin-4-yl)ethene (2/1) and 4-*n*-but­oxy­benzoic acid–(*E*)-1,2-di(pyridin-4-yl)ethene (2/1)

**DOI:** 10.1107/S2056989016017138

**Published:** 2016-10-28

**Authors:** Yohei Tabuchi, Kazuma Gotoh, Hiroyuki Ishida

**Affiliations:** a Department of Chemistry, Faculty of Science, Okayama University, Okayama 700-8530, Japan

**Keywords:** crystal structure, (*E*)-1,2-di(pyridin-4-yl)ethene, 4-alk­oxy­benzoic acid, hydrogen-bonded liquid crystal

## Abstract

The crystal structures of four hydrogen-bonded co-crystals of 4-alk­oxy­benzoic acid–(*E*)-1,2-di(pyridin-4-yl)ethene (2/1), namely, 2C_8_H_8_O_3_·C_12_H_10_N_2_, (I), 2C_9_H_10_O_3_·C_12_H_10_N_2_, (II), 2C_10_H_12_O_3_·C_12_H_10_N_2_, (III) and 2C_11_H_14_O_3_·C_12_H_10_N_2_, (IV), have been determined at 93 K. In compounds (I) and (IV), the asymmetric units are each composed of one 4-alk­oxy­benzoic acid mol­ecule and one half-mol­ecule of (*E*)-1,2-di(pyridin-4-yl)ethene, which lies on an inversion centre. The asymmetric unit of (II) consists of two crystallographically independent 4-eth­oxy­benzoic acid mol­ecules and one 1,2-di(pyridin-4-yl)ethene mol­ecule. Compound (III) crystallizes in a non-centrosymmetric space group (*Pc*) and the asymmetric unit comprises four 4-*n*-propoxybenzoic acid mol­ecules and two (*E*)-1,2-di(pyridin-4-yl)ethane mol­ecules. In each crystal, the acid and base components are linked by O—H⋯N hydrogen bonds, forming a linear hydrogen-bonded 2:1 unit of the acid and the base. In (I), (II) and (III), inter­molecular C—H⋯O inter­actions are observed. The 2:1 units of (I) and (II) are linked *via* C—H⋯O hydrogen bonds, forming tape structures. In (III), the C—H⋯O hydrogen bonds, except for those formed in the units, link the two crystallographically independent 2:1 units. In (IV), no C—H⋯O inter­actions are observed, but π–π and C—H⋯π inter­actions link the units into a column structure.

## Abstract 2

Crystal structures of four co-crystals of (*E*)-1,2-di(pyridin-4-yl)ethene with 4-alk­oxy­benzoic acids have been determined. Each compound comprises two acid mol­ecules and one base mol­ecule, which are held together by O—H⋯N hydrogen bonds, forming a linear hydrogen-bonded 2:1 unit.

## Chemical context   

Co-crystals of 4-alk­oxy­benzoic acid–4,4′-bipyridyl (2/1) and 4-alk­oxy­benzoic acid–(*E*)-1,2-di(pyridin-4-yl)ethene [common name: *trans*-1,2-bis­(4-pyrid­yl)ethyl­ene] (2/1), in which the two acids and the base are held together by hydrogen bonds, exhibit thermotropic liquid crystallinity (Kato *et al.*, 1990 ▸, 1993 ▸; Grunert *et al.*, 1997 ▸). Similar co-crystals of 4-alk­oxy­benzoic acid–1,2-bis­(pyridin-4-yl)ethane (2/1) also show thermotropic liquid crystallinity, namely, nematic phases at 419, 421 and 419 K for the compounds of 4-meth­oxy-, 4-eth­oxy- and 4-*n*-propoxybenzoic acid, respectively, and a smectic A phase at 413 K and a nematic phase at 419 K for the compound of 4-*n*-but­oxy­benzoic acid (Tabuchi *et al.*, 2015*a* ▸). The crystal structures of the compound of 4,4′-bipyridyl with 4-meth­oxy­benzoic acid (Mukherjee & Desiraju, 2014 ▸; Ramon *et al.*, 2014 ▸), the three compounds of 4,4′-bipyridyl with 4-eth­oxy-, 4-*n*-prop­oxy- and 4-*n*-but­oxy­benzoic acid (Tabuchi *et al.*, 2015*b* ▸), the compound of 1,2-bis­(pyridin-4-yl)ethane with 4-meth­oxy­benzoic acid (Mukherjee & Desiraju, 2014 ▸) and the three compounds of 1,2-bis­(pyridin-4-yl)ethane with with 4-eth­oxy-, 4-*n*-prop­oxy- and 4-*n*-but­oxy­benzoic acid (Tabuchi *et al.*, 2015*a* ▸) have been reported. As an expansion of our work on the structural characterization of hydrogen-bonded co-crystals which exhibit liquid phases, we have prepared four compounds of 4-alk­oxy­benzoic acid–(*E*)-1,2-di(pyridin-4-yl)ethene (2/1) and analyzed the crystal structures.
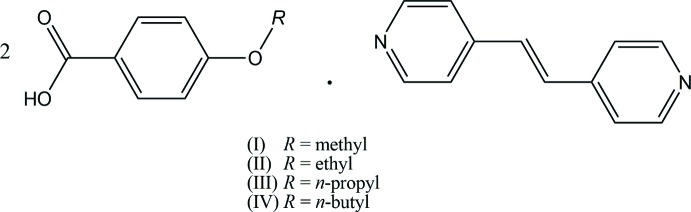


## Structural commentary   

**Figure 1:**
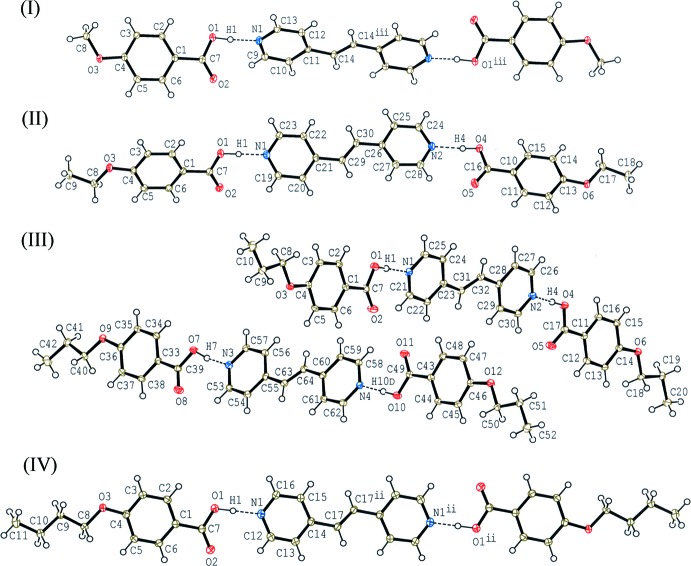
The mol­ecular structures of compounds (I), (II), (III) and (IV) determined at 93 K, showing the atom-numbering scheme. Displacement ellipsoids of non-H atoms are drawn at the 50% probability level and H atoms are drawn as circles of arbitrary size. The O—H⋯N hydrogen bonds are indicated by dashed lines. [Symmetry code for (I): (iii) −*x* + 1, −*y* + 1, −*z*; symmetry code for (IV): (ii) −*x* + 1, −*y* + 1, −*z* + 1.]

The mol­ecular structures of compounds (I)–(IV) are shown in Fig. 1 ▸. The asymmetric units of (I) and (IV) are each composed of one 4-alk­oxy­benzoic acid mol­ecule and one half-mol­ecule of (*E*)-1,2-di(pyridin-4-yl)ethene, which lies on an inversion centre. The two acid mol­ecules and the base mol­ecule are held together *via* O—H⋯N hydrogen bonds (Tables 1 ▸ and 4 ▸) to afford a centrosymmetric linear 2:1 unit. The hydrogen-bonded asymmetric unit of (I) is twisted with dihedral angles of 48.93 (12), 8.66 (12) and 57.16 (5)°, respectively, between the pyridine (N1/C9–C13) and carboxyl (O1/C7/O2) planes, the carboxyl and benzene (C1–C6) planes, and the pyridine and benzene rings, while the asymmetric unit of (IV) is approximately planar with dihedral angles of 5.24 (11), 3.29 (11) and 8.36 (4)°, respectively, between the pyridine (N1/C12–C16) and carboxyl (O1/C7/O2) planes, the carboxyl and benzene (C1–C6) planes, and the pyridine and benzene rings.

The asymmetric unit of (II) consists of two crystallographically independent 4-eth­oxy­benzoic acid mol­ecules and one (*E*)-1,2-di(pyridin-4-yl)ethene mol­ecule, and the two acids and the base are held together by O—H⋯N hydrogen bonds (Table 2 ▸), forming a linear hydrogen-bonded 2:1 aggregate. The pyridine rings of the base mol­ecule are twisted slightly to each other with a dihedral angle of 11.61 (5)°. One side of the hydrogen-bonded unit, *i.e.* C1–C7/O1/O2/N1/C19–C23, is considerably twisted, while the other side, *i.e.* C10–C16/O4/O5/N2/C24–C28, is approximately planar, which causes an additional C—H⋯O inter­action (C28—H28⋯O5; Table 2 ▸) between the acid and the base. The dihedral angles between the benzene (C1–C6) and pyridine (N1/C19–C23) rings, the C1–C6 and carboxyl O1/C7/O2 planes, and the N1/C19–C23 and O1/C7/O2 planes are 50.52 (5), 6.68 (13) and 43.98 (13)°, respectively, while the corresponding angles for the other side are 6.12 (5), 3.00 (12) and 3.38 (12)°, respectively, between the C10–C15 and N2/C24–C28 rings, the C10–C15 and carboxyl O4/C16/O5 planes, and the N2/C24–C28 and O4/C16/O5 planes.

The asymmetric unit of (III) is composed of four crystallographically independent mol­ecules of 4-*n*-propoxybenzoic acid and two base mol­ecules, forming two independent linear hydrogen-bonded 2:1 aggregates through O—H⋯N hydrogen bonds (Table 3 ▸). Both of the independent base mol­ecules are essentially planar with dihedral angles of 1.84 (8) and 0.58 (7)°, respectively, between the pyridine N1/C21–C25 and N2/C26–C30 rings, and between the pyridine N3/C53–C57 and N4/C58–C62 rings. The two hydrogen-bonded 2:1 units are also approximately planar. For one unit, the dihedral angles between the N1/C21–C25 and O1/C7/O2 planes, the C1–C6 and O1/C7/O2 planes, and the N1/C21–C25 and C1–C6 planes are 12.79 (19), 3.66 (19) and 9.16 (7)°, respectively, and the corresponding angles between the N2/C26–C30 and O4/C17/O5 planes, the C11–C16 and O4/C17/O5 planes, and the N2/C26–C30 and C11–C16 planes are 5.95 (19), 1.16 (19) and 5.82 (8)°, respectively. For the other 2:1 unit, the dihedral angles are 3.19 (19), 4.93 (19) and 7.59 (8)°, respectively, between the N3/C53–C57 and O7/C39/O8 planes, the C33–C38 and O7/C39/O8 planes, and the N3/C53–C57 and C33–C38 planes, and the corresponding dihedral angles are 7.71 (19), 7.70 (19) and 15.40 (8)°, respectively, between the N4/C58–C62 and O10/C49/O11 planes, the C43–C48 and O10/C49/O11 planes, and the N4/C58–C62 and C43–C48 planes.

## Supra­molecular features   

**Figure 2:**
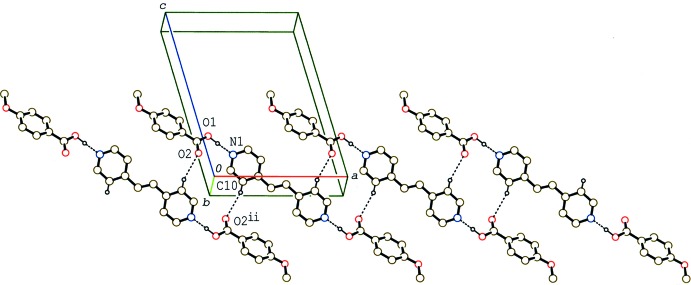
A partial packing diagram of compound (I), showing the tape structure formed by C—H⋯O and O—H⋯N hydrogen bonds (dashed lines). H atoms not involved in the hydrogen bonds have been omitted. [Symmetry code: (ii) −*x*, −*y* + 1, −*z*.]

In the crystal of (I), the 2:1 units are linked into a tape structure along the *a* axis through a pair of C—H⋯O hydrogen bonds (C10—H10⋯O2^ii^; symmetry code in Table 1 ▸), forming an 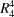
(16) ring motif together with O—H⋯N hydrogen bonds (Fig. 2 ▸). In addition, another C—H⋯O hydrogen bond (C8—H8*B*⋯O2^i^; symmetry code in Table 1 ▸) links the tapes into a three-dimensional network.

**Figure 3:**
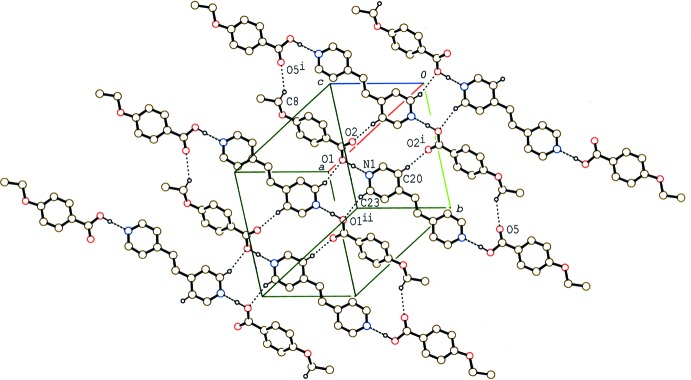
A partial packing diagram of compound (II), showing the tape structure formed by C—H⋯O and O—H⋯N hydrogen bonds (dashed lines). H atoms not involved in the hydrogen bonds have been omitted. [Symmetry codes: (i) −*x*, −*y* + 1, −*z* + 1; (ii) −*x* + 1, −*y* + 1, −*z* + 1.]

In the crystal of (II), the 2:1 units are linked by C—H⋯O inter­actions (C8—H8*A*⋯O5^i^, C20—H20⋯O2^i^ and C23—H23⋯O1^ii^; symmetry codes in Table 2 ▸), forming a tape structure along the *a* axis (Fig. 3 ▸). Between the tapes, another C—H⋯O and a C—H⋯π inter­action (C24—H24⋯O2^iii^ and C8—H8*B*⋯*Cg* ^iv^; *Cg* is the centroid of the C10–C15 benzene ring; Table 2 ▸) are observed.

**Figure 4:**
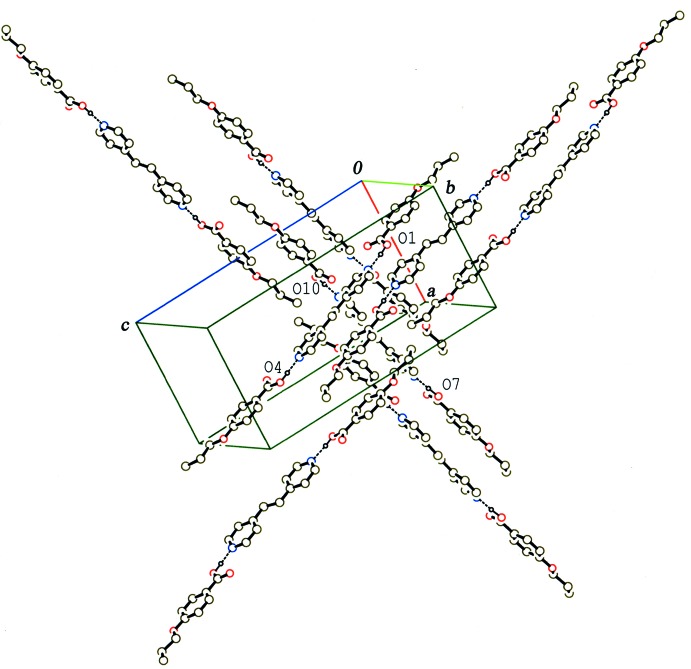
A partial packing diagram of compound (III), showing the two independent layers. H atoms not involved in the O—H⋯N hydrogen bonds (dashed lines) have been omitted.

In the crystal of (III), two crystallographically independent 2:1 units separately form layers parallel to the *ac* plane through weak C—H⋯π inter­actions (Table 3 ▸). These two layers are alternately stacked along the *b* axis through the C—H⋯O inter­actions (Table 3 ▸ and Fig. 4 ▸). In each layer, the 2:1 units are arranged with their long axes parallel to each other, while the units in neighbouring layers are arranged approximately perpendicular with an angle of *ca* 87° between their long axes (Fig. 4 ▸).

**Figure 5:**
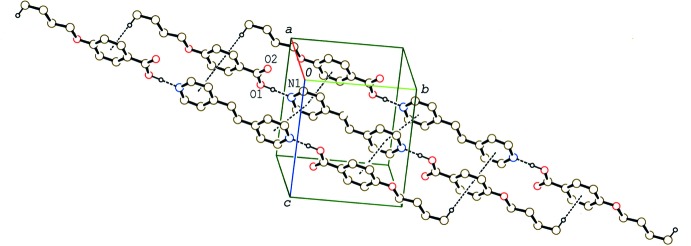
A partial packing diagram of compound (IV), showing the column structure formed by π–π and C—H⋯π inter­actions (dashed lines). H atoms not involved in these inter­actions have been omitted.

In the crystal of (IV), the 2:1units are stacked in a column along the *b* axis through a weak C—H⋯π inter­action between the methyl group and the benzene ring (Table 4 ▸) and π–π inter­actions between the benzene (C1–C6) and pyridine (N1/C12–C16) rings and between the pyridine rings (Fig. 5 ▸). The centroid–centroid distances are 3.658 (2) and 3.960 (2) Å, respectively, between the benzene and pyridine rings and between the pyridine rings.

## Database survey   

A search of the Cambridge Structural Database (Version 5.37, last update May 2016; Groom *et al.*, 2016 ▸) for organic co-crystals of 1,2-di(pyridin-4-yl)ethene with 4-alk­oxy­benzoic acid derivatives gave two structures: 1,2-di(pyridin-4-yl)ethene with 2,4,6-tris­(4-carb­oxy­phen­oxy)-1,3,5-triazine (Refcode YAKVEM; Aakeröy *et al.*, 2005 ▸) and with 4,4′-oxydi­benzoic acid (Refcode QEWHEH; Ma *et al.*, 2006 ▸).

## Synthesis and crystallization   

Single crystals of compounds (I), (III) and (IV) were obtained from ethanol solutions of (*E*)-1,2-di(pyridin-4-yl)ethene with 4-meth­oxy­benzoic acid, 4-*n*-propoxybenzoic acid and 4-*n*-but­oxy­benzoic acid, respectively, at room temperature [ethanol solution (160 ml) of 1,2-di(pyridin-4-yl)ethene (72 mg) and 4-meth­oxy­benzoic acid (120 mg) for (I), ethanol solution (160 ml) of 1,2-di(pyridin-4-yl)ethene (61 mg) and 4-*n*-propoxybenzoic acid (120 mg) for (III), and ethanol solution (160 ml) of 1,2-di(pyridin-4-yl)ethene (56 mg) and 4-*n*-but­oxy­benzoic acid (120 mg) for (IV)]. Crystals of compound (II) were obtained by slow evaporation from an acetone solution (150 ml) of 1,2-di(pyridin-4-yl)ethene (66 mg) with 4-eth­oxy­benzoic acid (120 mg) at room temperature.

## Phase transitions   

Phase transitions of the four title compounds were observed by DSC and the liquid crystal phases were confirmed by polarizing microscope. DSC measurements were performed by using a Perkin Elmer Pyris 1 in the temperature range from 103 K to the melting temperature at a heating rate of 10 K min^−1^. Phase transition temperatures (K) and enthalpies (kJ mol^−1^) determined by DSC are as follows:

(I) 439.0 (7) [60 (3)] K → N, 457.3 (5) [4.0 (2)] N → I;

(II) 432.6 (5) [66.6 (17)] K → N, 461 (1) [6.8 (15)] N → I;

(III) 401.0 (6) [16.5 (10)] K_1_ → K_2_, 425.2 (5) [45.6 (13)] K_2_ → N, 450.2 (5) [5.0 (5)] N → I;

(IV) 417.5 (5) [65 (2)] K → S_A_, 438 (1) [1.4 (2)] S_A_ → N, 449 (1) [6.1 (10)] N → I.

K, S_A_, N and I denote crystal, smectic A, nematic and isotropic phases, respectively. The observed transition temperatures and enthalpies are good agreement with the reported values (Kato *et al.*, 1993 ▸).

## Refinement   

Crystal data, data collection and structure refinement details are summarized in Table 5 ▸. For all compounds, C-bound H atoms were positioned geometrically with C—H = 0.95–0.99 Å and were refined as riding with *U*_iso_(H) = 1.2*U*_eq_(C) or 1.5*U*_eq_(methyl C). The O-bound H atoms were located in a difference Fourier map and refined freely [refined O—H = 0.93 (2)–1.02 (2) Å].

## Supplementary Material

Crystal structure: contains datablock(s) General, I, II, III, IV. DOI: 10.1107/S2056989016017138/lh5826sup1.cif

Structure factors: contains datablock(s) I. DOI: 10.1107/S2056989016017138/lh5826Isup2.hkl

Structure factors: contains datablock(s) II. DOI: 10.1107/S2056989016017138/lh5826IIsup3.hkl

Structure factors: contains datablock(s) III. DOI: 10.1107/S2056989016017138/lh5826IIIsup4.hkl

Structure factors: contains datablock(s) IV. DOI: 10.1107/S2056989016017138/lh5826IVsup5.hkl

Supporting information file. DOI: 10.1107/S2056989016017138/lh5826Isup6.cml

Supporting information file. DOI: 10.1107/S2056989016017138/lh5826IIsup7.cml

Supporting information file. DOI: 10.1107/S2056989016017138/lh5826IIIsup8.cml

Supporting information file. DOI: 10.1107/S2056989016017138/lh5826IVsup9.cml

CCDC references: 1511411, 1511410, 1511409, 1511408

Additional supporting information:  crystallographic information; 3D view; checkCIF report

## Figures and Tables

**Table 1:** Hydrogen-bond geometry (Å, °) for (I)

*D*—H⋯*A*	*D*—H	H⋯*A*	*D*⋯*A*	*D*—H⋯*A*
O1—H1⋯N1	1.01 (2)	1.61 (2)	2.6210 (15)	175.0 (17)
C8—H8*B*⋯O2^i^	0.98	2.56	3.4993 (18)	162
C10—H10⋯O2^ii^	0.95	2.57	3.4900 (18)	162

Symmetry codes: (i) 
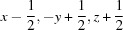
; (ii) 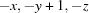.

**Table 2:** Hydrogen-bond geometry (Å, °) for (II) *Cg* is the centroid of the C10–C15 benzene ring.

*D*—H⋯*A*	*D*—H	H⋯*A*	*D*⋯*A*	*D*—H⋯*A*
O1—H1⋯N1	1.02 (2)	1.57 (2)	2.5931 (14)	178 (3)
O4—H4⋯N2	0.93 (2)	1.76 (2)	2.6858 (15)	177 (2)
C8—H8*A*⋯O5^i^	0.99	2.50	3.316 (2)	139
C20—H20⋯O2^i^	0.95	2.29	3.238 (2)	173
C23—H23⋯O1^ii^	0.95	2.58	3.449 (2)	152
C24—H24⋯O2^iii^	0.95	2.47	3.2993 (17)	146
C28—H28⋯O5	0.95	2.56	3.2291 (19)	128
C8—H8*B*⋯*Cg* ^iv^	0.00	2.75	3.6471 (18)	150

Symmetry codes: (i) 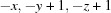; (ii) 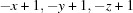; (iii) 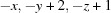; (iv) 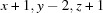.

**Table 3:** Hydrogen-bond geometry (Å, °) for (III) *Cg*1, *Cg*2, *Cg*3 and *Cg*4 are the centroids of the N1/C21–C25 pyridine, C1–C6 benzene, C11–C16 benzene and N4/C58–C62 pyridine rings, respectively.

*D*—H⋯*A*	*D*—H	H⋯*A*	*D*⋯*A*	*D*—H⋯*A*
O1—H1⋯N1	0.95 (4)	1.71 (4)	2.6607 (19)	175 (4)
O4—H4⋯N2	0.98 (3)	1.62 (3)	2.5974 (19)	179 (4)
O7—H7⋯N3	1.01 (2)	1.59 (2)	2.5836 (18)	170 (3)
O10—H10*D*⋯N4	0.97 (3)	1.63 (3)	2.5950 (18)	179 (3)
C21—H21⋯O2	0.95	2.50	3.193 (2)	130
C21—H21⋯O11	0.95	2.50	3.124 (2)	123
C26—H26⋯O7^i^	0.95	2.55	3.236 (2)	129
C30—H30⋯O8^ii^	0.95	2.47	3.155 (2)	129
C57—H57⋯O2^iii^	0.95	2.45	3.146 (2)	130
C58—H58⋯O11	0.95	2.51	3.165 (2)	126
C62—H62⋯O5^iv^	0.95	2.37	3.057 (2)	129
C8—H8*A*⋯*Cg*1^v^	0.99	2.85	3.6843 (19)	143
C8—H8*B*⋯*Cg*3^vi^	0.99	2.83	3.7013 (19)	147
C18—H18*A*⋯*Cg*2^vii^	0.99	2.80	3.5897 (19)	137
C50—H50*B*⋯*Cg*4^viii^	0.99	2.79	3.5721 (18)	136

Symmetry codes: (i) 
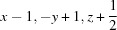
; (ii) 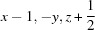; (iii) 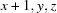; (iv) 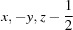; (v) 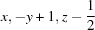; (vi) 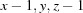; (vii) 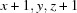; (viii) 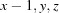.

**Table 4:** Hydrogen-bond geometry (Å, °) for (IV) *Cg* is the centroid of the C1–C6 benzene ring.

*D*—H⋯*A*	*D*—H	H⋯*A*	*D*⋯*A*	*D*—H⋯*A*
O1—H1⋯N1	1.02 (3)	1.57 (3)	2.5912 (18)	179 (2)
C11—H11*C*⋯*Cg* ^i^	0.98	2.92	3.800 (2)	150

Symmetry code: (i) 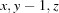.

**Table 5:** Experimental details

	(I)	(II)	(III)	(IV)
Crystal data				
Chemical formula	2C_8_H_8_O_3_·C_12_H_10_N_2_	2C_9_H_10_O_3_·C_12_H_10_N_2_	2C_10_H_12_O_3_·C_12_H_10_N_2_	2C_11_H_14_O_3_·C_12_H_10_N_2_
*M*_r_	486.52	514.58	542.63	570.68
Crystal system, space group	Monoclinic, *P*2_1_/*n*	Triclinic, *P* 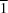	Monoclinic, *P* *c*	Triclinic, *P* 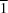
Temperature (K)	93	93	93	93
*a*, *b*, *c* (Å)	11.259 (4), 7.2693 (17), 14.758 (4)	10.873 (3), 11.197 (4), 12.921 (4)	11.1192 (18), 10.8289 (13), 23.020 (3)	7.103 (4), 9.060 (5), 11.627 (7)
α, β, γ (°)	90, 105.706 (15), 90	82.399 (13), 66.241 (10), 62.207 (11)	90, 93.517 (8), 90	82.29 (2), 78.54 (3), 86.79 (3)
*V* (Å^3^)	1162.8 (6)	1270.6 (7)	2766.6 (7)	726.3 (7)
*Z*	2	2	4	1
Radiation type	Mo *K*α	Mo *K*α	Mo *K*α	Mo *K*α
μ (mm^−1^)	0.10	0.09	0.09	0.09
Crystal size (mm)	0.31 × 0.30 × 0.10	0.40 × 0.13 × 0.10	0.47 × 0.27 × 0.10	0.49 × 0.21 × 0.10

Data collection				
Diffractometer	Rigaku R-AXIS RAPIDII	Rigaku R-AXIS RAPIDII	Rigaku R-AXIS RAPIDII	Rigaku R-AXIS RAPIDII
Absorption correction	Multi-scan (*ABSCOR*; Higashi, 1995 ▸)	Multi-scan (*ABSCOR*; Higashi, 1995 ▸)	Multi-scan (*ABSCOR*; Higashi, 1995 ▸)	Multi-scan (*ABSCOR*; Higashi, 1995 ▸)
*T*_min_, *T*_max_	0.896, 0.990	0.900, 0.991	0.914, 0.991	0.841, 0.991
No. of measured, independent and observed [*I* > 2σ(*I*)] reflections	11262, 2674, 2437	20685, 5814, 5127	43672, 11868, 11427	7250, 3311, 2919
*R*_int_	0.015	0.015	0.022	0.033
(sin θ/λ)_max_ (Å^−1^)	0.649	0.649	0.649	0.649

Refinement				
*R*[*F* ^2^ > 2σ(*F* ^2^)], *wR*(*F* ^2^), *S*	0.037, 0.099, 1.10	0.038, 0.109, 1.09	0.032, 0.080, 1.06	0.041, 0.118, 1.08
No. of reflections	2674	5814	11868	3311
No. of parameters	168	353	742	195
No. of restraints	0	0	2	0
H-atom treatment	H atoms treated by a mixture of independent and constrained refinement	H atoms treated by a mixture of independent and constrained refinement	H atoms treated by a mixture of independent and constrained refinement	H atoms treated by a mixture of independent and constrained refinement
Δρ_max_, Δρ_min_ (e Å^−3^)	0.29, −0.30	0.30, −0.35	0.19, −0.45	0.18, −0.45
Absolute structure	–	–	Refined as an inversion twin.	–
Absolute structure parameter	–	–	0.0 (5)	–

Computer programs: *RAPID-AUTO* (Rigaku, 2006 ▸), *Il Milione* (Burla *et al.*, 2007 ▸), *SHELXL2014* (Sheldrick, 2015 ▸), *ORTEP-3 for Windows* (Farrugia, 2012 ▸), *CrystalStructure* (Rigaku, 2010 ▸) and *PLATON* (Spek, 2015 ▸).

## supplementary crystallographic information

### (I) 4-Methoxybenzoic acid–(E)-1,2-di(pyridin-4-yl)ethene (2/1) Crystal data

**Table:**

2C_8_H_8_O_3_·C_12_H_10_N_2_	*F*(000) = 512.00
*M**_r_* = 486.52	*D*_x_ = 1.389 Mg m^−^^3^
Monoclinic, *P*2_1_/*n*	Mo *K*α radiation, λ = 0.71075 Å
Hall symbol: -P 2yn	Cell parameters from 12695 reflections
*a* = 11.259 (4) Å	θ = 3.2–30.1°
*b* = 7.2693 (17) Å	µ = 0.10 mm^−^^1^
*c* = 14.758 (4) Å	*T* = 93 K
β = 105.706 (15)°	Block, colorless
*V* = 1162.8 (6) Å^3^	0.31 × 0.30 × 0.10 mm
*Z* = 2	

### (I) 4-Methoxybenzoic acid–(E)-1,2-di(pyridin-4-yl)ethene (2/1) Data collection

**Table:**

Rigaku R-AXIS RAPIDII diffractometer	2437 reflections with *I* > 2σ(*I*)
Detector resolution: 10.000 pixels mm^-1^	*R*_int_ = 0.015
ω scans	θ_max_ = 27.5°, θ_min_ = 3.2°
Absorption correction: multi-scan (*ABSCOR*; Higashi, 1995)	*h* = −14→14
*T*_min_ = 0.896, *T*_max_ = 0.990	*k* = −9→8
11262 measured reflections	*l* = −19→19
2674 independent reflections	

### (I) 4-Methoxybenzoic acid–(E)-1,2-di(pyridin-4-yl)ethene (2/1) Refinement

**Table:**

Refinement on *F*^2^	Primary atom site location: structure-invariant direct methods
Least-squares matrix: full	Secondary atom site location: difference Fourier map
*R*[*F*^2^ > 2σ(*F*^2^)] = 0.037	Hydrogen site location: mixed
*wR*(*F*^2^) = 0.099	H atoms treated by a mixture of independent and constrained refinement
*S* = 1.10	*w* = 1/[σ^2^(*F*_o_^2^) + (0.0554*P*)^2^ + 0.3311*P*] where *P* = (*F*_o_^2^ + 2*F*_c_^2^)/3
2674 reflections	(Δ/σ)_max_ < 0.001
168 parameters	Δρ_max_ = 0.29 e Å^−^^3^
0 restraints	Δρ_min_ = −0.30 e Å^−^^3^

### (I) 4-Methoxybenzoic acid–(E)-1,2-di(pyridin-4-yl)ethene (2/1) Special details

**Table:**

Geometry. All esds (except the esd in the dihedral angle between two l.s. planes) are estimated using the full covariance matrix. The cell esds are taken into account individually in the estimation of esds in distances, angles and torsion angles; correlations between esds in cell parameters are only used when they are defined by crystal symmetry. An approximate (isotropic) treatment of cell esds is used for estimating esds involving l.s. planes.

### (I) 4-Methoxybenzoic acid–(E)-1,2-di(pyridin-4-yl)ethene (2/1) Fractional atomic coordinates and isotropic or equivalent isotropic displacement parameters (Å^2^)

**Table:**

	*x*	*y*	*z*	*U*_iso_*/*U*_eq_	
O1	0.07932 (7)	0.45344 (11)	0.29896 (5)	0.02040 (18)	
O2	−0.04269 (7)	0.29971 (11)	0.17718 (5)	0.02025 (18)	
O3	−0.38497 (6)	0.33362 (10)	0.45442 (5)	0.01748 (17)	
N1	0.22710 (8)	0.49461 (12)	0.19037 (6)	0.0198 (2)	
C1	−0.11584 (8)	0.36124 (13)	0.31108 (6)	0.0137 (2)	
C2	−0.10070 (8)	0.46264 (13)	0.39320 (7)	0.0143 (2)	
H2	−0.0288	0.5358	0.4154	0.017*	
C3	−0.18891 (9)	0.45914 (13)	0.44362 (6)	0.0146 (2)	
H3	−0.1775	0.5289	0.4998	0.017*	
C4	−0.29405 (8)	0.35178 (13)	0.41034 (7)	0.0143 (2)	
C5	−0.31129 (9)	0.25306 (14)	0.32684 (7)	0.0173 (2)	
H5	−0.3842	0.1825	0.3036	0.021*	
C6	−0.22277 (9)	0.25724 (14)	0.27769 (7)	0.0166 (2)	
H6	−0.2349	0.1890	0.2210	0.020*	
C7	−0.02356 (9)	0.36617 (13)	0.25543 (7)	0.0148 (2)	
C8	−0.36144 (10)	0.40882 (15)	0.54742 (7)	0.0194 (2)	
H8A	−0.2817	0.3640	0.5861	0.029*	
H8B	−0.4269	0.3705	0.5757	0.029*	
H8C	−0.3597	0.5434	0.5441	0.029*	
C9	0.17790 (9)	0.54944 (14)	0.10152 (7)	0.0196 (2)	
H9	0.0931	0.5823	0.0829	0.023*	
C10	0.24464 (9)	0.56063 (14)	0.03539 (7)	0.0177 (2)	
H10	0.2061	0.6007	−0.0269	0.021*	
C11	0.36946 (9)	0.51212 (13)	0.06160 (7)	0.0155 (2)	
C12	0.42043 (9)	0.45621 (14)	0.15457 (7)	0.0179 (2)	
H12	0.5051	0.4229	0.1756	0.022*	
C13	0.34688 (10)	0.44970 (15)	0.21567 (7)	0.0195 (2)	
H13	0.3830	0.4115	0.2787	0.023*	
C14	0.44046 (9)	0.52048 (14)	−0.00820 (7)	0.0171 (2)	
H14	0.3980	0.5582	−0.0701	0.021*	
H1	0.1344 (19)	0.463 (3)	0.2554 (14)	0.064 (6)*	

### (I) 4-Methoxybenzoic acid–(E)-1,2-di(pyridin-4-yl)ethene (2/1) Atomic displacement parameters (Å^2^)

**Table:**

	*U*^11^	*U*^22^	*U*^33^	*U*^12^	*U*^13^	*U*^23^
O1	0.0160 (4)	0.0290 (4)	0.0188 (4)	−0.0048 (3)	0.0093 (3)	−0.0038 (3)
O2	0.0205 (4)	0.0259 (4)	0.0163 (3)	0.0010 (3)	0.0083 (3)	−0.0027 (3)
O3	0.0145 (3)	0.0221 (4)	0.0183 (3)	−0.0021 (3)	0.0087 (3)	−0.0021 (3)
N1	0.0206 (4)	0.0212 (4)	0.0215 (4)	−0.0045 (3)	0.0123 (3)	−0.0041 (3)
C1	0.0136 (4)	0.0150 (4)	0.0132 (4)	0.0024 (3)	0.0047 (3)	0.0026 (3)
C2	0.0118 (4)	0.0170 (4)	0.0138 (4)	0.0001 (3)	0.0028 (3)	0.0014 (3)
C3	0.0144 (4)	0.0174 (4)	0.0120 (4)	0.0013 (3)	0.0038 (3)	−0.0005 (3)
C4	0.0129 (4)	0.0157 (4)	0.0155 (4)	0.0022 (3)	0.0063 (3)	0.0027 (3)
C5	0.0157 (4)	0.0171 (5)	0.0194 (5)	−0.0031 (4)	0.0054 (4)	−0.0021 (4)
C6	0.0183 (5)	0.0170 (5)	0.0151 (4)	−0.0006 (4)	0.0055 (3)	−0.0023 (4)
C7	0.0157 (4)	0.0147 (4)	0.0148 (4)	0.0027 (3)	0.0054 (3)	0.0024 (3)
C8	0.0207 (5)	0.0228 (5)	0.0179 (5)	−0.0004 (4)	0.0106 (4)	−0.0013 (4)
C9	0.0163 (5)	0.0207 (5)	0.0237 (5)	−0.0013 (4)	0.0089 (4)	−0.0033 (4)
C10	0.0178 (5)	0.0183 (5)	0.0180 (4)	−0.0017 (4)	0.0066 (4)	−0.0014 (4)
C11	0.0170 (5)	0.0149 (4)	0.0167 (4)	−0.0037 (4)	0.0081 (4)	−0.0029 (4)
C12	0.0165 (5)	0.0216 (5)	0.0172 (5)	−0.0017 (4)	0.0070 (4)	−0.0011 (4)
C13	0.0214 (5)	0.0228 (5)	0.0160 (4)	−0.0032 (4)	0.0083 (4)	−0.0013 (4)
C14	0.0188 (5)	0.0198 (5)	0.0146 (4)	−0.0023 (4)	0.0076 (3)	−0.0007 (4)

### (I) 4-Methoxybenzoic acid–(E)-1,2-di(pyridin-4-yl)ethene (2/1) Geometric parameters (Å, º)

**Table:**

O1—C7	1.3244 (12)	C5—H5	0.9500
O1—H1	1.01 (2)	C6—H6	0.9500
O2—C7	1.2160 (12)	C8—H8A	0.9800
O3—C4	1.3599 (11)	C8—H8B	0.9800
O3—C8	1.4338 (12)	C8—H8C	0.9800
N1—C9	1.3385 (14)	C9—C10	1.3859 (14)
N1—C13	1.3388 (15)	C9—H9	0.9500
C1—C2	1.3889 (14)	C10—C11	1.3981 (15)
C1—C6	1.3938 (14)	C10—H10	0.9500
C1—C7	1.4890 (13)	C11—C12	1.3967 (14)
C2—C3	1.3931 (13)	C11—C14	1.4661 (13)
C2—H2	0.9500	C12—C13	1.3804 (14)
C3—C4	1.3917 (14)	C12—H12	0.9500
C3—H3	0.9500	C13—H13	0.9500
C4—C5	1.3934 (14)	C14—C14^i^	1.330 (2)
C5—C6	1.3826 (14)	C14—H14	0.9500
			
C7—O1—H1	109.4 (11)	O3—C8—H8A	109.5
C4—O3—C8	116.93 (8)	O3—C8—H8B	109.5
C9—N1—C13	117.66 (9)	H8A—C8—H8B	109.5
C2—C1—C6	119.12 (9)	O3—C8—H8C	109.5
C2—C1—C7	121.96 (9)	H8A—C8—H8C	109.5
C6—C1—C7	118.88 (9)	H8B—C8—H8C	109.5
C1—C2—C3	121.30 (9)	N1—C9—C10	123.22 (10)
C1—C2—H2	119.4	N1—C9—H9	118.4
C3—C2—H2	119.4	C10—C9—H9	118.4
C4—C3—C2	118.88 (9)	C9—C10—C11	119.11 (9)
C4—C3—H3	120.6	C9—C10—H10	120.4
C2—C3—H3	120.6	C11—C10—H10	120.4
O3—C4—C3	124.35 (9)	C12—C11—C10	117.39 (9)
O3—C4—C5	115.49 (9)	C12—C11—C14	123.02 (9)
C3—C4—C5	120.16 (9)	C10—C11—C14	119.58 (9)
C6—C5—C4	120.33 (9)	C13—C12—C11	119.47 (10)
C6—C5—H5	119.8	C13—C12—H12	120.3
C4—C5—H5	119.8	C11—C12—H12	120.3
C5—C6—C1	120.18 (9)	N1—C13—C12	123.14 (10)
C5—C6—H6	119.9	N1—C13—H13	118.4
C1—C6—H6	119.9	C12—C13—H13	118.4
O2—C7—O1	123.90 (9)	C14^i^—C14—C11	125.27 (12)
O2—C7—C1	122.92 (9)	C14^i^—C14—H14	117.4
O1—C7—C1	113.16 (8)	C11—C14—H14	117.4
			
C6—C1—C2—C3	1.38 (14)	C6—C1—C7—O2	8.04 (14)
C7—C1—C2—C3	178.85 (8)	C2—C1—C7—O1	9.13 (13)
C1—C2—C3—C4	−0.14 (14)	C6—C1—C7—O1	−173.39 (8)
C8—O3—C4—C3	−9.37 (13)	C13—N1—C9—C10	0.34 (15)
C8—O3—C4—C5	170.34 (9)	N1—C9—C10—C11	0.23 (15)
C2—C3—C4—O3	178.32 (8)	C9—C10—C11—C12	−0.64 (14)
C2—C3—C4—C5	−1.37 (14)	C9—C10—C11—C14	178.99 (9)
O3—C4—C5—C6	−178.08 (9)	C10—C11—C12—C13	0.50 (14)
C3—C4—C5—C6	1.64 (15)	C14—C11—C12—C13	−179.11 (9)
C4—C5—C6—C1	−0.37 (15)	C9—N1—C13—C12	−0.49 (15)
C2—C1—C6—C5	−1.12 (14)	C11—C12—C13—N1	0.07 (16)
C7—C1—C6—C5	−178.67 (8)	C12—C11—C14—C14^i^	−1.01 (19)
C2—C1—C7—O2	−169.44 (9)	C10—C11—C14—C14^i^	179.39 (13)

Symmetry code: (i) −*x*+1, −*y*+1, −*z*.

### (I) 4-Methoxybenzoic acid–(E)-1,2-di(pyridin-4-yl)ethene (2/1) Hydrogen-bond geometry (Å, º)

**Table:**

*D*—H···*A*	*D*—H	H···*A*	*D*···*A*	*D*—H···*A*
O1—H1···N1	1.01 (2)	1.61 (2)	2.6210 (15)	175.0 (17)
C8—H8*B*···O2^ii^	0.98	2.56	3.4993 (18)	162
C10—H10···O2^iii^	0.95	2.57	3.4900 (18)	162

Symmetry codes: (ii) *x*−1/2, −*y*+1/2, *z*+1/2; (iii) −*x*, −*y*+1, −*z*.

### (II) 4-Ethoxybenzoic acid–(E)-1,2-di(pyridin-4-yl)ethene (2/1) Crystal data

**Table:**

2C_9_H_10_O_3_·C_12_H_10_N_2_	*Z* = 2
*M**_r_* = 514.58	*F*(000) = 544.00
Triclinic, *P*1	*D*_x_ = 1.345 Mg m^−^^3^
Hall symbol: -P 1	Mo *K*α radiation, λ = 0.71075 Å
*a* = 10.873 (3) Å	Cell parameters from 22362 reflections
*b* = 11.197 (4) Å	θ = 3.3–30.0°
*c* = 12.921 (4) Å	µ = 0.09 mm^−^^1^
α = 82.399 (13)°	*T* = 93 K
β = 66.241 (10)°	Platelet, colorless
γ = 62.207 (11)°	0.40 × 0.13 × 0.10 mm
*V* = 1270.6 (7) Å^3^	

### (II) 4-Ethoxybenzoic acid–(E)-1,2-di(pyridin-4-yl)ethene (2/1) Data collection

**Table:**

Rigaku R-AXIS RAPIDII diffractometer	5127 reflections with *I* > 2σ(*I*)
Detector resolution: 10.000 pixels mm^-1^	*R*_int_ = 0.015
ω scans	θ_max_ = 27.5°, θ_min_ = 3.3°
Absorption correction: multi-scan (*ABSCOR*; Higashi, 1995)	*h* = −14→14
*T*_min_ = 0.900, *T*_max_ = 0.991	*k* = −14→14
20685 measured reflections	*l* = −16→16
5814 independent reflections	

### (II) 4-Ethoxybenzoic acid–(E)-1,2-di(pyridin-4-yl)ethene (2/1) Refinement

**Table:**

Refinement on *F*^2^	Primary atom site location: structure-invariant direct methods
Least-squares matrix: full	Secondary atom site location: difference Fourier map
*R*[*F*^2^ > 2σ(*F*^2^)] = 0.038	Hydrogen site location: mixed
*wR*(*F*^2^) = 0.109	H atoms treated by a mixture of independent and constrained refinement
*S* = 1.09	*w* = 1/[σ^2^(*F*_o_^2^) + (0.0717*P*)^2^ + 0.1676*P*] where *P* = (*F*_o_^2^ + 2*F*_c_^2^)/3
5814 reflections	(Δ/σ)_max_ = 0.001
353 parameters	Δρ_max_ = 0.30 e Å^−^^3^
0 restraints	Δρ_min_ = −0.35 e Å^−^^3^

### (II) 4-Ethoxybenzoic acid–(E)-1,2-di(pyridin-4-yl)ethene (2/1) Special details

**Table:**

Geometry. All esds (except the esd in the dihedral angle between two l.s. planes) are estimated using the full covariance matrix. The cell esds are taken into account individually in the estimation of esds in distances, angles and torsion angles; correlations between esds in cell parameters are only used when they are defined by crystal symmetry. An approximate (isotropic) treatment of cell esds is used for estimating esds involving l.s. planes.

### (II) 4-Ethoxybenzoic acid–(E)-1,2-di(pyridin-4-yl)ethene (2/1) Fractional atomic coordinates and isotropic or equivalent isotropic displacement parameters (Å^2^)

**Table:**

	*x*	*y*	*z*	*U*_iso_*/*U*_eq_	
O1	0.44575 (8)	0.29817 (7)	0.49920 (6)	0.02270 (17)	
O2	0.22493 (8)	0.30835 (7)	0.62196 (6)	0.02228 (16)	
O3	0.70637 (8)	−0.25170 (7)	0.71620 (6)	0.01917 (15)	
O4	−0.40769 (8)	1.64915 (7)	0.18066 (6)	0.02136 (16)	
O5	−0.50193 (9)	1.52880 (7)	0.14118 (7)	0.02571 (17)	
O6	−0.87480 (8)	2.10135 (7)	−0.00980 (6)	0.01967 (16)	
N1	0.28738 (9)	0.53158 (8)	0.43973 (7)	0.01887 (18)	
N2	−0.24387 (9)	1.41152 (8)	0.24761 (7)	0.01975 (18)	
C1	0.45252 (10)	0.11559 (9)	0.61365 (8)	0.01602 (19)	
C2	0.60886 (11)	0.04391 (10)	0.55614 (8)	0.01699 (19)	
H2	0.6599	0.0802	0.4926	0.020*	
C3	0.68971 (10)	−0.07954 (10)	0.59126 (8)	0.01692 (19)	
H3	0.7958	−0.1281	0.5513	0.020*	
C4	0.61597 (10)	−0.13298 (9)	0.68521 (8)	0.01573 (18)	
C5	0.45923 (11)	−0.06414 (10)	0.74142 (8)	0.01780 (19)	
H5	0.4078	−0.1014	0.8039	0.021*	
C6	0.37964 (10)	0.05957 (10)	0.70471 (8)	0.01762 (19)	
H6	0.2731	0.1067	0.7428	0.021*	
C7	0.36266 (11)	0.24964 (9)	0.57911 (8)	0.01740 (19)	
C8	0.63496 (12)	−0.30580 (10)	0.81677 (9)	0.0216 (2)	
H8A	0.5750	−0.3412	0.8019	0.026*	
H8B	0.5663	−0.2340	0.8791	0.026*	
C9	0.75677 (12)	−0.41802 (10)	0.84936 (9)	0.0231 (2)	
H9A	0.8258	−0.4873	0.7863	0.035*	
H9B	0.7109	−0.4584	0.9163	0.035*	
H9C	0.8129	−0.3813	0.8667	0.035*	
C10	−0.59976 (10)	1.76311 (9)	0.10913 (8)	0.01658 (19)	
C11	−0.69670 (11)	1.75879 (10)	0.06566 (8)	0.0195 (2)	
H11	−0.6993	1.6759	0.0617	0.023*	
C12	−0.78868 (11)	1.87368 (10)	0.02853 (8)	0.0189 (2)	
H12	−0.8551	1.8699	0.0003	0.023*	
C13	−0.78373 (10)	1.99537 (9)	0.03265 (8)	0.01663 (19)	
C14	−0.68928 (11)	2.00189 (10)	0.07724 (8)	0.01755 (19)	
H14	−0.6868	2.0848	0.0812	0.021*	
C15	−0.59854 (11)	1.88556 (10)	0.11586 (8)	0.01718 (19)	
H15	−0.5351	1.8900	0.1471	0.021*	
C16	−0.49962 (11)	1.63572 (10)	0.14515 (8)	0.01805 (19)	
C17	−0.86841 (12)	2.22730 (10)	−0.01373 (9)	0.0206 (2)	
H17A	−0.7653	2.2150	−0.0621	0.025*	
H17B	−0.8948	2.2603	0.0634	0.025*	
C18	−0.97968 (12)	2.32750 (10)	−0.06207 (9)	0.0258 (2)	
H18A	−1.0820	2.3430	−0.0111	0.039*	
H18B	−0.9557	2.2915	−0.1366	0.039*	
H18C	−0.9737	2.4131	−0.0699	0.039*	
C19	0.16443 (11)	0.55222 (10)	0.42448 (8)	0.0193 (2)	
H19	0.1384	0.4801	0.4358	0.023*	
C20	0.07334 (11)	0.67374 (10)	0.39300 (8)	0.01813 (19)	
H20	−0.0128	0.6840	0.3829	0.022*	
C21	0.10966 (10)	0.78120 (9)	0.37619 (8)	0.01547 (19)	
C22	0.23808 (11)	0.75891 (10)	0.39243 (8)	0.01754 (19)	
H22	0.2670	0.8290	0.3820	0.021*	
C23	0.32276 (11)	0.63424 (10)	0.42375 (8)	0.0191 (2)	
H23	0.4097	0.6207	0.4344	0.023*	
C24	−0.13435 (11)	1.39130 (10)	0.28095 (8)	0.01879 (19)	
H24	−0.1176	1.4660	0.2858	0.023*	
C25	−0.04417 (11)	1.26610 (10)	0.30882 (8)	0.01838 (19)	
H25	0.0332	1.2562	0.3310	0.022*	
C26	−0.06734 (10)	1.15489 (9)	0.30414 (8)	0.01639 (19)	
C27	−0.18146 (12)	1.17653 (10)	0.26908 (9)	0.0234 (2)	
H27	−0.2012	1.1039	0.2638	0.028*	
C28	−0.26563 (12)	1.30443 (11)	0.24211 (10)	0.0252 (2)	
H28	−0.3428	1.3171	0.2185	0.030*	
C29	0.01377 (10)	0.91023 (9)	0.34238 (8)	0.01701 (19)	
H29	−0.0630	0.9128	0.3231	0.020*	
C30	0.02655 (10)	1.02405 (10)	0.33681 (8)	0.01731 (19)	
H30	0.1041	1.0200	0.3559	0.021*	
H1	0.382 (3)	0.391 (2)	0.4775 (19)	0.082 (7)*	
H4	−0.354 (2)	1.566 (2)	0.2045 (15)	0.059 (5)*	

### (II) 4-Ethoxybenzoic acid–(E)-1,2-di(pyridin-4-yl)ethene (2/1) Atomic displacement parameters (Å^2^)

**Table:**

	*U*^11^	*U*^22^	*U*^33^	*U*^12^	*U*^13^	*U*^23^
O1	0.0197 (3)	0.0169 (3)	0.0299 (4)	−0.0070 (3)	−0.0119 (3)	0.0082 (3)
O2	0.0170 (3)	0.0157 (3)	0.0329 (4)	−0.0046 (3)	−0.0118 (3)	0.0011 (3)
O3	0.0174 (3)	0.0149 (3)	0.0216 (3)	−0.0047 (3)	−0.0086 (3)	0.0048 (3)
O4	0.0205 (3)	0.0162 (3)	0.0307 (4)	−0.0079 (3)	−0.0152 (3)	0.0069 (3)
O5	0.0339 (4)	0.0152 (3)	0.0356 (4)	−0.0118 (3)	−0.0216 (3)	0.0078 (3)
O6	0.0211 (3)	0.0140 (3)	0.0269 (4)	−0.0073 (3)	−0.0145 (3)	0.0066 (3)
N1	0.0182 (4)	0.0156 (4)	0.0194 (4)	−0.0053 (3)	−0.0076 (3)	0.0032 (3)
N2	0.0182 (4)	0.0158 (4)	0.0223 (4)	−0.0052 (3)	−0.0089 (3)	0.0038 (3)
C1	0.0168 (4)	0.0131 (4)	0.0199 (4)	−0.0057 (3)	−0.0096 (4)	−0.0002 (3)
C2	0.0177 (4)	0.0164 (4)	0.0177 (4)	−0.0079 (4)	−0.0078 (4)	0.0018 (3)
C3	0.0137 (4)	0.0162 (4)	0.0187 (4)	−0.0047 (3)	−0.0063 (3)	−0.0004 (3)
C4	0.0173 (4)	0.0121 (4)	0.0190 (4)	−0.0052 (3)	−0.0098 (4)	0.0003 (3)
C5	0.0176 (4)	0.0166 (4)	0.0194 (4)	−0.0086 (4)	−0.0066 (4)	0.0017 (3)
C6	0.0144 (4)	0.0161 (4)	0.0214 (4)	−0.0056 (3)	−0.0068 (4)	−0.0014 (3)
C7	0.0186 (4)	0.0138 (4)	0.0224 (4)	−0.0063 (4)	−0.0113 (4)	−0.0002 (3)
C8	0.0227 (5)	0.0180 (5)	0.0252 (5)	−0.0107 (4)	−0.0105 (4)	0.0075 (4)
C9	0.0285 (5)	0.0172 (5)	0.0256 (5)	−0.0091 (4)	−0.0151 (4)	0.0055 (4)
C10	0.0165 (4)	0.0148 (4)	0.0171 (4)	−0.0065 (3)	−0.0068 (4)	0.0037 (3)
C11	0.0229 (5)	0.0160 (4)	0.0230 (5)	−0.0111 (4)	−0.0100 (4)	0.0038 (4)
C12	0.0203 (4)	0.0187 (5)	0.0221 (4)	−0.0104 (4)	−0.0114 (4)	0.0045 (4)
C13	0.0151 (4)	0.0150 (4)	0.0173 (4)	−0.0055 (3)	−0.0063 (4)	0.0033 (3)
C14	0.0186 (4)	0.0142 (4)	0.0210 (4)	−0.0083 (4)	−0.0083 (4)	0.0027 (3)
C15	0.0169 (4)	0.0166 (4)	0.0188 (4)	−0.0079 (4)	−0.0078 (4)	0.0027 (3)
C16	0.0183 (4)	0.0157 (4)	0.0184 (4)	−0.0072 (4)	−0.0067 (4)	0.0029 (3)
C17	0.0243 (5)	0.0135 (4)	0.0241 (5)	−0.0075 (4)	−0.0117 (4)	0.0037 (4)
C18	0.0286 (5)	0.0161 (5)	0.0288 (5)	−0.0046 (4)	−0.0152 (4)	0.0046 (4)
C19	0.0209 (5)	0.0146 (4)	0.0222 (4)	−0.0081 (4)	−0.0083 (4)	0.0021 (3)
C20	0.0163 (4)	0.0164 (4)	0.0217 (4)	−0.0066 (4)	−0.0083 (4)	0.0005 (3)
C21	0.0155 (4)	0.0128 (4)	0.0146 (4)	−0.0043 (3)	−0.0052 (3)	0.0009 (3)
C22	0.0181 (4)	0.0158 (4)	0.0194 (4)	−0.0084 (4)	−0.0077 (4)	0.0036 (3)
C23	0.0168 (4)	0.0191 (5)	0.0208 (4)	−0.0072 (4)	−0.0087 (4)	0.0042 (4)
C24	0.0202 (5)	0.0146 (4)	0.0203 (4)	−0.0072 (4)	−0.0079 (4)	0.0019 (3)
C25	0.0183 (4)	0.0168 (4)	0.0205 (4)	−0.0070 (4)	−0.0095 (4)	0.0022 (3)
C26	0.0155 (4)	0.0142 (4)	0.0159 (4)	−0.0049 (3)	−0.0053 (3)	0.0022 (3)
C27	0.0257 (5)	0.0164 (5)	0.0348 (5)	−0.0106 (4)	−0.0185 (5)	0.0076 (4)
C28	0.0244 (5)	0.0208 (5)	0.0366 (6)	−0.0105 (4)	−0.0194 (5)	0.0086 (4)
C29	0.0147 (4)	0.0149 (4)	0.0191 (4)	−0.0046 (3)	−0.0077 (4)	0.0031 (3)
C30	0.0157 (4)	0.0151 (4)	0.0190 (4)	−0.0043 (3)	−0.0085 (4)	0.0025 (3)

### (II) 4-Ethoxybenzoic acid–(E)-1,2-di(pyridin-4-yl)ethene (2/1) Geometric parameters (Å, º)

**Table:**

O1—C7	1.3173 (12)	C11—H11	0.9500
O1—H1	1.02 (2)	C12—C13	1.3972 (14)
O2—C7	1.2213 (12)	C12—H12	0.9500
O3—C4	1.3606 (11)	C13—C14	1.3963 (13)
O3—C8	1.4407 (12)	C14—C15	1.3948 (13)
O4—C16	1.3267 (12)	C14—H14	0.9500
O4—H4	0.93 (2)	C15—H15	0.9500
O5—C16	1.2173 (13)	C17—C18	1.5067 (14)
O6—C13	1.3636 (12)	C17—H17A	0.9900
O6—C17	1.4373 (12)	C17—H17B	0.9900
N1—C19	1.3385 (13)	C18—H18A	0.9800
N1—C23	1.3406 (13)	C18—H18B	0.9800
N2—C24	1.3384 (13)	C18—H18C	0.9800
N2—C28	1.3413 (14)	C19—C20	1.3853 (13)
C1—C6	1.3866 (13)	C19—H19	0.9500
C1—C2	1.3978 (14)	C20—C21	1.3985 (14)
C1—C7	1.4877 (13)	C20—H20	0.9500
C2—C3	1.3826 (13)	C21—C22	1.3977 (13)
C2—H2	0.9500	C21—C29	1.4698 (13)
C3—C4	1.3948 (13)	C22—C23	1.3829 (13)
C3—H3	0.9500	C22—H22	0.9500
C4—C5	1.3970 (14)	C23—H23	0.9500
C5—C6	1.3899 (14)	C24—C25	1.3876 (13)
C5—H5	0.9500	C24—H24	0.9500
C6—H6	0.9500	C25—C26	1.3937 (14)
C8—C9	1.5060 (14)	C25—H25	0.9500
C8—H8A	0.9900	C26—C27	1.3954 (14)
C8—H8B	0.9900	C26—C30	1.4682 (13)
C9—H9A	0.9800	C27—C28	1.3830 (14)
C9—H9B	0.9800	C27—H27	0.9500
C9—H9C	0.9800	C28—H28	0.9500
C10—C15	1.3917 (14)	C29—C30	1.3347 (14)
C10—C11	1.4002 (14)	C29—H29	0.9500
C10—C16	1.4904 (13)	C30—H30	0.9500
C11—C12	1.3816 (14)		
			
C7—O1—H1	112.5 (13)	C10—C15—C14	120.80 (9)
C4—O3—C8	117.16 (8)	C10—C15—H15	119.6
C16—O4—H4	107.3 (12)	C14—C15—H15	119.6
C13—O6—C17	117.81 (8)	O5—C16—O4	123.33 (9)
C19—N1—C23	117.86 (8)	O5—C16—C10	122.47 (9)
C24—N2—C28	117.29 (8)	O4—C16—C10	114.19 (8)
C6—C1—C2	118.97 (9)	O6—C17—C18	107.58 (8)
C6—C1—C7	119.35 (9)	O6—C17—H17A	110.2
C2—C1—C7	121.68 (9)	C18—C17—H17A	110.2
C3—C2—C1	120.38 (9)	O6—C17—H17B	110.2
C3—C2—H2	119.8	C18—C17—H17B	110.2
C1—C2—H2	119.8	H17A—C17—H17B	108.5
C2—C3—C4	120.20 (9)	C17—C18—H18A	109.5
C2—C3—H3	119.9	C17—C18—H18B	109.5
C4—C3—H3	119.9	H18A—C18—H18B	109.5
O3—C4—C3	115.67 (8)	C17—C18—H18C	109.5
O3—C4—C5	124.38 (8)	H18A—C18—H18C	109.5
C3—C4—C5	119.94 (9)	H18B—C18—H18C	109.5
C6—C5—C4	119.07 (9)	N1—C19—C20	123.14 (9)
C6—C5—H5	120.5	N1—C19—H19	118.4
C4—C5—H5	120.5	C20—C19—H19	118.4
C1—C6—C5	121.38 (9)	C19—C20—C21	119.28 (9)
C1—C6—H6	119.3	C19—C20—H20	120.4
C5—C6—H6	119.3	C21—C20—H20	120.4
O2—C7—O1	123.82 (9)	C22—C21—C20	117.24 (8)
O2—C7—C1	122.55 (9)	C22—C21—C29	123.22 (8)
O1—C7—C1	113.62 (8)	C20—C21—C29	119.54 (9)
O3—C8—C9	108.07 (8)	C23—C22—C21	119.66 (9)
O3—C8—H8A	110.1	C23—C22—H22	120.2
C9—C8—H8A	110.1	C21—C22—H22	120.2
O3—C8—H8B	110.1	N1—C23—C22	122.83 (9)
C9—C8—H8B	110.1	N1—C23—H23	118.6
H8A—C8—H8B	108.4	C22—C23—H23	118.6
C8—C9—H9A	109.5	N2—C24—C25	122.96 (9)
C8—C9—H9B	109.5	N2—C24—H24	118.5
H9A—C9—H9B	109.5	C25—C24—H24	118.5
C8—C9—H9C	109.5	C24—C25—C26	119.90 (9)
H9A—C9—H9C	109.5	C24—C25—H25	120.1
H9B—C9—H9C	109.5	C26—C25—H25	120.1
C15—C10—C11	119.03 (9)	C25—C26—C27	116.87 (9)
C15—C10—C16	122.19 (9)	C25—C26—C30	119.11 (9)
C11—C10—C16	118.78 (9)	C27—C26—C30	124.01 (9)
C12—C11—C10	120.75 (9)	C28—C27—C26	119.58 (9)
C12—C11—H11	119.6	C28—C27—H27	120.2
C10—C11—H11	119.6	C26—C27—H27	120.2
C11—C12—C13	119.86 (9)	N2—C28—C27	123.40 (10)
C11—C12—H12	120.1	N2—C28—H28	118.3
C13—C12—H12	120.1	C27—C28—H28	118.3
O6—C13—C14	124.69 (9)	C30—C29—C21	124.82 (9)
O6—C13—C12	115.20 (8)	C30—C29—H29	117.6
C14—C13—C12	120.11 (9)	C21—C29—H29	117.6
C15—C14—C13	119.42 (9)	C29—C30—C26	126.47 (9)
C15—C14—H14	120.3	C29—C30—H30	116.8
C13—C14—H14	120.3	C26—C30—H30	116.8
			
C6—C1—C2—C3	−1.41 (14)	C13—C14—C15—C10	0.81 (14)
C7—C1—C2—C3	179.01 (8)	C15—C10—C16—O5	178.78 (9)
C1—C2—C3—C4	−0.64 (14)	C11—C10—C16—O5	−2.10 (14)
C8—O3—C4—C3	176.51 (8)	C15—C10—C16—O4	−1.78 (13)
C8—O3—C4—C5	−2.87 (13)	C11—C10—C16—O4	177.33 (8)
C2—C3—C4—O3	−176.95 (8)	C13—O6—C17—C18	−179.28 (8)
C2—C3—C4—C5	2.45 (14)	C23—N1—C19—C20	0.14 (14)
O3—C4—C5—C6	177.18 (8)	N1—C19—C20—C21	−0.13 (15)
C3—C4—C5—C6	−2.17 (14)	C19—C20—C21—C22	0.04 (14)
C2—C1—C6—C5	1.69 (14)	C19—C20—C21—C29	179.78 (8)
C7—C1—C6—C5	−178.72 (8)	C20—C21—C22—C23	0.04 (14)
C4—C5—C6—C1	0.10 (14)	C29—C21—C22—C23	−179.69 (9)
C6—C1—C7—O2	−6.00 (14)	C19—N1—C23—C22	−0.05 (14)
C2—C1—C7—O2	173.58 (9)	C21—C22—C23—N1	−0.04 (15)
C6—C1—C7—O1	173.62 (8)	C28—N2—C24—C25	0.33 (14)
C2—C1—C7—O1	−6.80 (13)	N2—C24—C25—C26	−0.90 (15)
C4—O3—C8—C9	−167.74 (8)	C24—C25—C26—C27	0.98 (14)
C15—C10—C11—C12	0.81 (14)	C24—C25—C26—C30	−178.40 (8)
C16—C10—C11—C12	−178.33 (9)	C25—C26—C27—C28	−0.58 (15)
C10—C11—C12—C13	0.96 (15)	C30—C26—C27—C28	178.77 (9)
C17—O6—C13—C14	2.93 (13)	C24—N2—C28—C27	0.09 (16)
C17—O6—C13—C12	−176.60 (8)	C26—C27—C28—N2	0.05 (17)
C11—C12—C13—O6	177.69 (8)	C22—C21—C29—C30	−8.51 (15)
C11—C12—C13—C14	−1.86 (14)	C20—C21—C29—C30	171.76 (9)
O6—C13—C14—C15	−178.53 (8)	C21—C29—C30—C26	−179.62 (8)
C12—C13—C14—C15	0.98 (14)	C25—C26—C30—C29	176.47 (9)
C11—C10—C15—C14	−1.70 (14)	C27—C26—C30—C29	−2.87 (16)
C16—C10—C15—C14	177.41 (8)		

### (II) 4-Ethoxybenzoic acid–(E)-1,2-di(pyridin-4-yl)ethene (2/1) Hydrogen-bond geometry (Å, º)

*Cg* is the centroid of the C10–C15 benzene ring.

**Table:**

*D*—H···*A*	*D*—H	H···*A*	*D*···*A*	*D*—H···*A*
O1—H1···N1	1.02 (2)	1.57 (2)	2.5931 (14)	178 (3)
O4—H4···N2	0.93 (2)	1.76 (2)	2.6858 (15)	177 (2)
C8—H8*A*···O5^i^	0.99	2.50	3.316 (2)	139
C20—H20···O2^i^	0.95	2.29	3.238 (2)	173
C23—H23···O1^ii^	0.95	2.58	3.449 (2)	152
C24—H24···O2^iii^	0.95	2.47	3.2993 (17)	146
C28—H28···O5	0.95	2.56	3.2291 (19)	128
C8—H8*B*···*Cg*^iv^	0.00	2.75	3.6471 (18)	150

Symmetry codes: (i) −*x*, −*y*+1, −*z*+1; (ii) −*x*+1, −*y*+1, −*z*+1; (iii) −*x*, −*y*+2, −*z*+1; (iv) *x*+1, *y*−2, *z*+1.

### (III) 4-n-Propoxybenzoic acid–(E)-1,2-di(pyridin-4-yl)ethene (2/1) Crystal data

**Table:**

2C_10_H_12_O_3_·C_12_H_10_N_2_	*F*(000) = 1152.00
*M**_r_* = 542.63	*D*_x_ = 1.303 Mg m^−^^3^
Monoclinic, *P**c*	Mo *K*α radiation, λ = 0.71075 Å
Hall symbol: P -2yc	Cell parameters from 52745 reflections
*a* = 11.1192 (18) Å	θ = 3.1–30.1°
*b* = 10.8289 (13) Å	µ = 0.09 mm^−^^1^
*c* = 23.020 (3) Å	*T* = 93 K
β = 93.517 (8)°	Block, colorless
*V* = 2766.6 (7) Å^3^	0.47 × 0.27 × 0.10 mm
*Z* = 4	

### (III) 4-n-Propoxybenzoic acid–(E)-1,2-di(pyridin-4-yl)ethene (2/1) Data collection

**Table:**

Rigaku R-AXIS RAPIDII diffractometer	11427 reflections with *I* > 2σ(*I*)
Detector resolution: 10.000 pixels mm^-1^	*R*_int_ = 0.022
ω scans	θ_max_ = 27.5°, θ_min_ = 3.1°
Absorption correction: multi-scan (*ABSCOR*; Higashi, 1995)	*h* = −14→14
*T*_min_ = 0.914, *T*_max_ = 0.991	*k* = −13→14
43672 measured reflections	*l* = −29→29
11868 independent reflections	

### (III) 4-n-Propoxybenzoic acid–(E)-1,2-di(pyridin-4-yl)ethene (2/1) Refinement

**Table:**

Refinement on *F*^2^	Secondary atom site location: difference Fourier map
Least-squares matrix: full	Hydrogen site location: mixed
*R*[*F*^2^ > 2σ(*F*^2^)] = 0.032	H atoms treated by a mixture of independent and constrained refinement
*wR*(*F*^2^) = 0.080	*w* = 1/[σ^2^(*F*_o_^2^) + (0.0601*P*)^2^ + 0.1097*P*] where *P* = (*F*_o_^2^ + 2*F*_c_^2^)/3
*S* = 1.06	(Δ/σ)_max_ = 0.001
11868 reflections	Δρ_max_ = 0.19 e Å^−^^3^
742 parameters	Δρ_min_ = −0.45 e Å^−^^3^
2 restraints	Absolute structure: Refined as an inversion twin.
Primary atom site location: structure-invariant direct methods	Absolute structure parameter: 0.0 (5)

### (III) 4-n-Propoxybenzoic acid–(E)-1,2-di(pyridin-4-yl)ethene (2/1) Special details

**Table:**

Geometry. All esds (except the esd in the dihedral angle between two l.s. planes) are estimated using the full covariance matrix. The cell esds are taken into account individually in the estimation of esds in distances, angles and torsion angles; correlations between esds in cell parameters are only used when they are defined by crystal symmetry. An approximate (isotropic) treatment of cell esds is used for estimating esds involving l.s. planes.
Refinement. Refined as a 2-component inversion twin.

### (III) 4-n-Propoxybenzoic acid–(E)-1,2-di(pyridin-4-yl)ethene (2/1) Fractional atomic coordinates and isotropic or equivalent isotropic displacement parameters (Å^2^)

**Table:**

	*x*	*y*	*z*	*U*_iso_*/*U*_eq_	
O1	0.32953 (12)	0.53144 (11)	0.14865 (5)	0.0194 (2)	
O2	0.32547 (13)	0.32943 (11)	0.16857 (5)	0.0256 (3)	
O3	0.15985 (11)	0.32000 (10)	−0.09975 (5)	0.0175 (2)	
O4	0.74306 (11)	0.61561 (10)	0.76437 (5)	0.0192 (2)	
O5	0.74264 (12)	0.40996 (11)	0.75692 (5)	0.0246 (3)	
O6	0.90934 (11)	0.50197 (10)	1.02430 (5)	0.0170 (2)	
O7	1.64469 (10)	−0.01975 (11)	0.11593 (6)	0.0192 (2)	
O8	1.65201 (11)	−0.22435 (11)	0.10387 (6)	0.0231 (3)	
O9	2.16733 (10)	−0.04986 (11)	0.02766 (5)	0.0184 (2)	
O10	0.42885 (10)	−0.04174 (10)	0.31208 (6)	0.0189 (2)	
O11	0.44387 (11)	0.16385 (11)	0.31005 (6)	0.0232 (3)	
O12	−0.08488 (10)	0.10616 (10)	0.39427 (5)	0.0180 (2)	
N1	0.40817 (12)	0.55374 (13)	0.25966 (6)	0.0164 (3)	
N2	0.65872 (13)	0.61638 (12)	0.65662 (6)	0.0162 (3)	
N3	1.43371 (12)	−0.05632 (13)	0.15388 (6)	0.0161 (3)	
N4	0.64132 (12)	−0.02903 (12)	0.27170 (6)	0.0155 (3)	
C1	0.27225 (14)	0.39443 (14)	0.07180 (7)	0.0142 (3)	
C2	0.25007 (14)	0.49248 (14)	0.03367 (7)	0.0161 (3)	
H2	0.2613	0.5746	0.0474	0.019*	
C3	0.21176 (15)	0.47258 (14)	−0.02430 (7)	0.0164 (3)	
H3	0.1961	0.5404	−0.0499	0.020*	
C4	0.19672 (14)	0.35145 (14)	−0.04430 (7)	0.0146 (3)	
C5	0.21982 (14)	0.25211 (14)	−0.00639 (7)	0.0153 (3)	
H5	0.2102	0.1698	−0.0202	0.018*	
C6	0.25659 (14)	0.27355 (14)	0.05111 (7)	0.0155 (3)	
H6	0.2714	0.2058	0.0768	0.019*	
C7	0.31161 (14)	0.41411 (15)	0.13429 (7)	0.0159 (3)	
C8	0.13607 (15)	0.41737 (15)	−0.14122 (7)	0.0167 (3)	
H8A	0.2100	0.4662	−0.1461	0.020*	
H8B	0.0729	0.4732	−0.1279	0.020*	
C9	0.09421 (16)	0.35684 (16)	−0.19813 (7)	0.0204 (3)	
H9A	0.0196	0.3093	−0.1928	0.024*	
H9B	0.1567	0.2986	−0.2101	0.024*	
C10	0.07013 (17)	0.45360 (18)	−0.24547 (8)	0.0259 (4)	
H10A	0.0375	0.4133	−0.2812	0.039*	
H10B	0.1456	0.4957	−0.2531	0.039*	
H10C	0.0118	0.5140	−0.2326	0.039*	
C11	0.79737 (14)	0.50089 (14)	0.84888 (7)	0.0144 (3)	
C12	0.81679 (14)	0.38887 (14)	0.87723 (7)	0.0163 (3)	
H12	0.8040	0.3141	0.8561	0.020*	
C13	0.85464 (15)	0.38402 (14)	0.93601 (7)	0.0159 (3)	
H13	0.8679	0.3068	0.9549	0.019*	
C14	0.87278 (14)	0.49443 (15)	0.96683 (7)	0.0145 (3)	
C15	0.85349 (15)	0.60769 (14)	0.93858 (7)	0.0165 (3)	
H15	0.8663	0.6826	0.9595	0.020*	
C16	0.81583 (14)	0.61066 (14)	0.88022 (7)	0.0154 (3)	
H16	0.8024	0.6878	0.8613	0.019*	
C17	0.75811 (14)	0.50347 (14)	0.78582 (7)	0.0155 (3)	
C18	0.92950 (15)	0.38910 (14)	1.05584 (7)	0.0159 (3)	
H18A	0.9930	0.3400	1.0382	0.019*	
H18B	0.8546	0.3395	1.0547	0.019*	
C19	0.96841 (15)	0.42194 (15)	1.11810 (7)	0.0170 (3)	
H19A	0.9047	0.4715	1.1352	0.020*	
H19B	1.0427	0.4725	1.1188	0.020*	
C20	0.99190 (17)	0.30527 (16)	1.15432 (7)	0.0215 (3)	
H20A	1.0557	0.2567	1.1376	0.032*	
H20B	0.9180	0.2560	1.1542	0.032*	
H20C	1.0172	0.3283	1.1944	0.032*	
C21	0.44175 (14)	0.44775 (15)	0.28601 (7)	0.0172 (3)	
H21	0.4361	0.3734	0.2641	0.021*	
C22	0.48435 (14)	0.44187 (15)	0.34388 (7)	0.0162 (3)	
H22	0.5084	0.3649	0.3606	0.019*	
C23	0.49178 (14)	0.54936 (15)	0.37746 (7)	0.0146 (3)	
C24	0.45676 (14)	0.65969 (14)	0.34977 (7)	0.0162 (3)	
H24	0.4603	0.7353	0.3707	0.019*	
C25	0.41696 (15)	0.65777 (15)	0.29160 (7)	0.0170 (3)	
H25	0.3947	0.7337	0.2733	0.020*	
C26	0.62835 (14)	0.72313 (14)	0.63042 (7)	0.0163 (3)	
H26	0.6371	0.7973	0.6523	0.020*	
C27	0.58483 (14)	0.73036 (14)	0.57292 (7)	0.0157 (3)	
H27	0.5628	0.8080	0.5562	0.019*	
C28	0.57348 (14)	0.62230 (14)	0.53938 (7)	0.0143 (3)	
C29	0.60595 (15)	0.51143 (14)	0.56720 (7)	0.0163 (3)	
H29	0.6003	0.4358	0.5463	0.020*	
C30	0.64636 (15)	0.51207 (15)	0.62520 (7)	0.0175 (3)	
H30	0.6662	0.4356	0.6436	0.021*	
C31	0.53612 (14)	0.54115 (15)	0.43876 (7)	0.0157 (3)	
H31	0.5727	0.4657	0.4514	0.019*	
C32	0.52930 (14)	0.63108 (15)	0.47830 (7)	0.0151 (3)	
H32	0.4929	0.7066	0.4657	0.018*	
C33	1.82306 (14)	−0.10410 (14)	0.08278 (7)	0.0143 (3)	
C34	1.87138 (15)	0.01377 (14)	0.07642 (7)	0.0155 (3)	
H34	1.8251	0.0844	0.0851	0.019*	
C35	1.98640 (15)	0.02828 (14)	0.05759 (7)	0.0163 (3)	
H35	2.0184	0.1088	0.0530	0.020*	
C36	2.05535 (14)	−0.07502 (15)	0.04539 (7)	0.0155 (3)	
C37	2.00860 (15)	−0.19323 (15)	0.05190 (7)	0.0168 (3)	
H37	2.0552	−0.2638	0.0435	0.020*	
C38	1.89264 (14)	−0.20662 (14)	0.07082 (7)	0.0155 (3)	
H38	1.8606	−0.2871	0.0756	0.019*	
C39	1.69845 (14)	−0.12297 (14)	0.10180 (7)	0.0147 (3)	
C40	2.23943 (14)	−0.15240 (15)	0.01121 (7)	0.0169 (3)	
H40A	2.1987	−0.1966	−0.0221	0.020*	
H40B	2.2510	−0.2110	0.0441	0.020*	
C41	2.36007 (15)	−0.10366 (16)	−0.00547 (7)	0.0196 (3)	
H41A	2.4001	−0.0588	0.0278	0.024*	
H41B	2.3481	−0.0453	−0.0384	0.024*	
C42	2.43915 (17)	−0.21100 (18)	−0.02284 (9)	0.0282 (4)	
H42A	2.4542	−0.2663	0.0105	0.042*	
H42B	2.5160	−0.1790	−0.0352	0.042*	
H42C	2.3981	−0.2567	−0.0550	0.042*	
C43	0.26098 (14)	0.07523 (14)	0.33818 (7)	0.0148 (3)	
C44	0.20144 (14)	−0.03061 (14)	0.35473 (7)	0.0152 (3)	
H44	0.2407	−0.1083	0.3529	0.018*	
C45	0.08563 (14)	−0.02495 (14)	0.37387 (7)	0.0154 (3)	
H45	0.0460	−0.0980	0.3853	0.018*	
C46	0.02829 (14)	0.08912 (15)	0.37615 (7)	0.0149 (3)	
C47	0.08671 (15)	0.19663 (14)	0.35876 (7)	0.0183 (3)	
H47	0.0470	0.2742	0.3597	0.022*	
C48	0.20245 (15)	0.18907 (15)	0.34025 (7)	0.0183 (3)	
H48	0.2424	0.2619	0.3289	0.022*	
C49	0.38641 (14)	0.07066 (14)	0.31880 (7)	0.0151 (3)	
C50	−0.15245 (14)	−0.00148 (15)	0.40715 (7)	0.0162 (3)	
H50A	−0.1097	−0.0492	0.4387	0.019*	
H50B	−0.1626	−0.0549	0.3723	0.019*	
C51	−0.27412 (15)	0.04002 (15)	0.42582 (7)	0.0188 (3)	
H51A	−0.3160	0.0884	0.3942	0.023*	
H51B	−0.2632	0.0938	0.4605	0.023*	
C52	−0.34991 (16)	−0.07171 (17)	0.43997 (9)	0.0256 (4)	
H52A	−0.3600	−0.1250	0.4056	0.038*	
H52B	−0.4291	−0.0441	0.4513	0.038*	
H52C	−0.3095	−0.1179	0.4721	0.038*	
C53	1.37844 (15)	−0.16641 (14)	0.15381 (7)	0.0158 (3)	
H53	1.4217	−0.2375	0.1429	0.019*	
C54	1.26071 (14)	−0.18018 (14)	0.16912 (7)	0.0154 (3)	
H54	1.2243	−0.2596	0.1684	0.018*	
C55	1.19548 (14)	−0.07673 (14)	0.18562 (6)	0.0136 (3)	
C56	1.25420 (15)	0.03742 (14)	0.18563 (7)	0.0154 (3)	
H56	1.2136	0.1104	0.1963	0.018*	
C57	1.37192 (15)	0.04280 (15)	0.16993 (7)	0.0164 (3)	
H57	1.4111	0.1208	0.1705	0.020*	
C58	0.69887 (14)	0.07938 (14)	0.27061 (7)	0.0152 (3)	
H58	0.6572	0.1521	0.2807	0.018*	
C59	0.81687 (14)	0.08950 (14)	0.25528 (7)	0.0151 (3)	
H59	0.8551	0.1679	0.2554	0.018*	
C60	0.87958 (14)	−0.01604 (14)	0.23960 (6)	0.0139 (3)	
C61	0.81821 (14)	−0.12871 (14)	0.24027 (7)	0.0154 (3)	
H61	0.8570	−0.2029	0.2297	0.018*	
C62	0.70047 (14)	−0.13104 (15)	0.25645 (7)	0.0163 (3)	
H62	0.6597	−0.2081	0.2568	0.020*	
C63	1.07037 (14)	−0.09180 (14)	0.20120 (6)	0.0146 (3)	
H63	1.0336	−0.1701	0.1944	0.018*	
C64	1.00481 (14)	−0.00392 (14)	0.22417 (7)	0.0153 (3)	
H64	1.0426	0.0738	0.2312	0.018*	
H1	0.362 (4)	0.539 (4)	0.1878 (17)	0.086 (12)*	
H4	0.711 (3)	0.615 (3)	0.7237 (13)	0.050 (8)*	
H7	1.564 (2)	−0.044 (3)	0.1301 (11)	0.040 (7)*	
H10D	0.508 (3)	−0.036 (3)	0.2969 (14)	0.055 (9)*	

### (III) 4-n-Propoxybenzoic acid–(E)-1,2-di(pyridin-4-yl)ethene (2/1) Atomic displacement parameters (Å^2^)

**Table:**

	*U*^11^	*U*^22^	*U*^33^	*U*^12^	*U*^13^	*U*^23^
O1	0.0288 (6)	0.0148 (5)	0.0139 (5)	0.0000 (5)	−0.0040 (5)	−0.0016 (4)
O2	0.0432 (8)	0.0150 (5)	0.0174 (6)	−0.0006 (5)	−0.0085 (5)	0.0000 (4)
O3	0.0257 (6)	0.0137 (5)	0.0126 (5)	0.0002 (4)	−0.0019 (4)	0.0009 (4)
O4	0.0296 (6)	0.0137 (5)	0.0136 (5)	−0.0012 (5)	−0.0045 (5)	0.0009 (4)
O5	0.0400 (7)	0.0147 (5)	0.0181 (6)	−0.0005 (5)	−0.0064 (5)	−0.0021 (5)
O6	0.0237 (6)	0.0138 (5)	0.0129 (5)	0.0008 (4)	−0.0024 (5)	0.0003 (4)
O7	0.0151 (5)	0.0134 (5)	0.0300 (6)	0.0006 (4)	0.0069 (5)	−0.0004 (4)
O8	0.0206 (6)	0.0158 (5)	0.0333 (7)	−0.0029 (5)	0.0052 (5)	−0.0027 (5)
O9	0.0164 (5)	0.0157 (5)	0.0239 (6)	0.0012 (4)	0.0076 (5)	0.0001 (4)
O10	0.0148 (5)	0.0138 (5)	0.0286 (6)	0.0013 (4)	0.0061 (5)	0.0014 (5)
O11	0.0195 (6)	0.0141 (5)	0.0369 (7)	−0.0021 (5)	0.0090 (5)	−0.0015 (5)
O12	0.0155 (5)	0.0152 (5)	0.0237 (6)	−0.0002 (4)	0.0056 (5)	−0.0006 (4)
N1	0.0181 (6)	0.0164 (6)	0.0144 (6)	−0.0012 (5)	−0.0007 (5)	−0.0004 (5)
N2	0.0186 (6)	0.0159 (6)	0.0138 (6)	−0.0016 (5)	−0.0014 (5)	0.0000 (5)
N3	0.0143 (6)	0.0174 (6)	0.0167 (6)	0.0012 (5)	0.0011 (5)	0.0014 (5)
N4	0.0143 (6)	0.0152 (6)	0.0169 (6)	0.0008 (5)	0.0017 (5)	0.0009 (5)
C1	0.0139 (7)	0.0149 (7)	0.0138 (7)	0.0006 (5)	0.0006 (6)	−0.0007 (6)
C2	0.0183 (7)	0.0127 (7)	0.0172 (7)	−0.0006 (6)	0.0009 (6)	−0.0016 (6)
C3	0.0202 (8)	0.0116 (7)	0.0172 (7)	−0.0001 (5)	−0.0003 (6)	0.0025 (5)
C4	0.0137 (7)	0.0162 (7)	0.0139 (7)	−0.0003 (6)	0.0011 (6)	−0.0013 (6)
C5	0.0176 (7)	0.0102 (6)	0.0182 (7)	0.0004 (5)	0.0011 (6)	−0.0016 (6)
C6	0.0171 (7)	0.0131 (7)	0.0162 (7)	0.0007 (5)	−0.0001 (6)	0.0020 (6)
C7	0.0166 (7)	0.0162 (7)	0.0148 (7)	0.0006 (6)	−0.0007 (6)	−0.0012 (6)
C8	0.0195 (7)	0.0153 (7)	0.0150 (7)	0.0005 (6)	−0.0007 (6)	0.0026 (6)
C9	0.0236 (8)	0.0224 (8)	0.0149 (7)	0.0016 (6)	−0.0013 (6)	0.0004 (6)
C10	0.0276 (9)	0.0323 (9)	0.0175 (8)	0.0044 (7)	−0.0007 (7)	0.0053 (7)
C11	0.0143 (7)	0.0145 (7)	0.0143 (7)	−0.0005 (5)	−0.0003 (6)	0.0002 (6)
C12	0.0178 (7)	0.0134 (7)	0.0174 (7)	−0.0007 (6)	−0.0001 (6)	−0.0021 (6)
C13	0.0191 (7)	0.0117 (7)	0.0168 (7)	0.0001 (6)	−0.0001 (6)	0.0017 (6)
C14	0.0137 (7)	0.0162 (7)	0.0136 (7)	−0.0001 (5)	0.0000 (6)	0.0011 (5)
C15	0.0204 (7)	0.0127 (7)	0.0162 (7)	0.0000 (6)	−0.0009 (6)	−0.0021 (6)
C16	0.0178 (7)	0.0121 (7)	0.0162 (7)	0.0006 (6)	−0.0006 (6)	0.0007 (6)
C17	0.0152 (7)	0.0152 (7)	0.0159 (7)	−0.0011 (5)	−0.0002 (6)	0.0001 (6)
C18	0.0191 (8)	0.0143 (7)	0.0140 (7)	0.0011 (6)	−0.0006 (6)	0.0024 (6)
C19	0.0197 (8)	0.0179 (7)	0.0133 (7)	−0.0001 (6)	0.0001 (6)	0.0001 (6)
C20	0.0301 (9)	0.0189 (8)	0.0150 (7)	−0.0004 (6)	−0.0028 (7)	0.0002 (6)
C21	0.0193 (8)	0.0157 (7)	0.0166 (7)	−0.0017 (6)	0.0002 (6)	−0.0029 (6)
C22	0.0177 (7)	0.0140 (7)	0.0166 (7)	0.0006 (6)	−0.0001 (6)	0.0002 (6)
C23	0.0121 (7)	0.0169 (7)	0.0147 (7)	−0.0019 (5)	−0.0001 (6)	−0.0005 (6)
C24	0.0187 (8)	0.0140 (7)	0.0157 (7)	−0.0009 (6)	−0.0008 (6)	−0.0023 (6)
C25	0.0196 (8)	0.0143 (7)	0.0166 (7)	−0.0012 (6)	−0.0014 (6)	0.0013 (6)
C26	0.0191 (7)	0.0148 (7)	0.0148 (7)	−0.0005 (6)	0.0008 (6)	−0.0018 (6)
C27	0.0179 (7)	0.0133 (7)	0.0160 (7)	0.0014 (6)	0.0008 (6)	0.0012 (6)
C28	0.0123 (7)	0.0164 (7)	0.0141 (7)	−0.0018 (5)	0.0011 (6)	−0.0002 (6)
C29	0.0189 (7)	0.0136 (7)	0.0163 (7)	−0.0015 (6)	−0.0012 (6)	−0.0012 (6)
C30	0.0206 (8)	0.0142 (7)	0.0174 (8)	−0.0002 (6)	−0.0014 (6)	0.0025 (6)
C31	0.0166 (7)	0.0157 (7)	0.0145 (7)	−0.0012 (6)	−0.0010 (6)	0.0009 (6)
C32	0.0146 (7)	0.0155 (7)	0.0149 (7)	−0.0011 (5)	−0.0008 (6)	0.0016 (6)
C33	0.0155 (7)	0.0149 (7)	0.0126 (7)	−0.0006 (6)	0.0000 (6)	−0.0008 (5)
C34	0.0177 (7)	0.0134 (7)	0.0155 (7)	0.0017 (6)	0.0007 (6)	−0.0011 (5)
C35	0.0187 (8)	0.0128 (7)	0.0174 (7)	−0.0005 (6)	0.0021 (6)	−0.0005 (5)
C36	0.0157 (7)	0.0193 (8)	0.0113 (6)	−0.0004 (6)	0.0000 (6)	−0.0002 (6)
C37	0.0171 (7)	0.0143 (7)	0.0189 (7)	0.0025 (6)	0.0018 (6)	−0.0029 (6)
C38	0.0197 (8)	0.0108 (7)	0.0160 (7)	−0.0013 (6)	0.0000 (6)	−0.0011 (5)
C39	0.0158 (7)	0.0136 (7)	0.0146 (7)	0.0001 (6)	−0.0008 (6)	0.0003 (5)
C40	0.0163 (7)	0.0166 (7)	0.0180 (7)	0.0021 (6)	0.0024 (6)	−0.0015 (6)
C41	0.0175 (8)	0.0215 (8)	0.0202 (7)	0.0010 (6)	0.0041 (6)	0.0019 (6)
C42	0.0200 (8)	0.0284 (9)	0.0371 (10)	0.0055 (7)	0.0088 (8)	−0.0008 (8)
C43	0.0133 (7)	0.0157 (7)	0.0154 (7)	−0.0011 (6)	0.0007 (6)	−0.0013 (6)
C44	0.0159 (7)	0.0124 (7)	0.0171 (7)	0.0020 (5)	0.0000 (6)	0.0002 (5)
C45	0.0161 (7)	0.0138 (7)	0.0162 (7)	−0.0021 (6)	0.0002 (6)	0.0019 (5)
C46	0.0145 (7)	0.0166 (7)	0.0138 (7)	−0.0001 (6)	0.0015 (6)	−0.0011 (6)
C47	0.0184 (8)	0.0121 (7)	0.0247 (8)	0.0022 (6)	0.0034 (6)	−0.0007 (6)
C48	0.0177 (8)	0.0140 (7)	0.0233 (8)	−0.0026 (6)	0.0027 (6)	0.0007 (6)
C49	0.0159 (7)	0.0137 (7)	0.0156 (7)	0.0000 (5)	0.0012 (6)	−0.0001 (5)
C50	0.0171 (7)	0.0151 (7)	0.0167 (7)	−0.0019 (6)	0.0032 (6)	0.0007 (6)
C51	0.0173 (7)	0.0205 (8)	0.0190 (8)	−0.0007 (6)	0.0045 (6)	0.0002 (6)
C52	0.0198 (8)	0.0249 (9)	0.0327 (9)	−0.0034 (7)	0.0075 (7)	0.0030 (7)
C53	0.0162 (7)	0.0147 (7)	0.0165 (7)	0.0037 (6)	0.0017 (6)	0.0006 (6)
C54	0.0166 (7)	0.0144 (7)	0.0151 (7)	−0.0009 (6)	0.0003 (6)	0.0007 (5)
C55	0.0137 (7)	0.0167 (7)	0.0103 (6)	0.0008 (6)	0.0000 (5)	0.0014 (5)
C56	0.0164 (7)	0.0138 (7)	0.0160 (7)	0.0026 (6)	0.0015 (6)	0.0009 (5)
C57	0.0173 (7)	0.0147 (7)	0.0174 (7)	−0.0019 (6)	0.0020 (6)	0.0013 (6)
C58	0.0171 (7)	0.0136 (7)	0.0150 (7)	0.0027 (6)	0.0019 (6)	0.0011 (5)
C59	0.0173 (7)	0.0120 (7)	0.0160 (7)	−0.0008 (5)	0.0008 (6)	0.0012 (5)
C60	0.0144 (7)	0.0159 (7)	0.0113 (6)	0.0000 (6)	0.0000 (5)	0.0020 (5)
C61	0.0170 (7)	0.0128 (7)	0.0163 (7)	0.0021 (6)	0.0007 (6)	−0.0007 (5)
C62	0.0157 (8)	0.0148 (7)	0.0182 (7)	−0.0013 (5)	0.0007 (6)	0.0003 (6)
C63	0.0141 (7)	0.0154 (7)	0.0143 (7)	−0.0016 (6)	0.0000 (6)	0.0010 (5)
C64	0.0141 (7)	0.0156 (7)	0.0162 (7)	−0.0011 (6)	0.0012 (6)	0.0015 (6)

### (III) 4-n-Propoxybenzoic acid–(E)-1,2-di(pyridin-4-yl)ethene (2/1) Geometric parameters (Å, º)

**Table:**

O1—C7	1.325 (2)	C24—H24	0.9500
O1—H1	0.95 (4)	C25—H25	0.9500
O2—C7	1.214 (2)	C26—C27	1.383 (2)
O3—C4	1.3598 (18)	C26—H26	0.9500
O3—C8	1.4360 (18)	C27—C28	1.403 (2)
O4—C17	1.3177 (19)	C27—H27	0.9500
O4—H4	0.98 (3)	C28—C29	1.398 (2)
O5—C17	1.218 (2)	C28—C32	1.464 (2)
O6—C14	1.3625 (18)	C29—C30	1.383 (2)
O6—C18	1.4322 (18)	C29—H29	0.9500
O7—C39	1.3177 (19)	C30—H30	0.9500
O7—H7	1.01 (3)	C31—C32	1.338 (2)
O8—C39	1.215 (2)	C31—H31	0.9500
O9—C36	1.361 (2)	C32—H32	0.9500
O9—C40	1.4340 (18)	C33—C38	1.390 (2)
O10—C49	1.3180 (19)	C33—C34	1.396 (2)
O10—H10D	0.97 (3)	C33—C39	1.493 (2)
O11—C49	1.218 (2)	C34—C35	1.384 (2)
O12—C46	1.3624 (19)	C34—H34	0.9500
O12—C50	1.4278 (19)	C35—C36	1.394 (2)
N1—C21	1.340 (2)	C35—H35	0.9500
N1—C25	1.346 (2)	C36—C37	1.393 (2)
N2—C26	1.338 (2)	C37—C38	1.394 (2)
N2—C30	1.344 (2)	C37—H37	0.9500
N3—C57	1.339 (2)	C38—H38	0.9500
N3—C53	1.341 (2)	C40—C41	1.513 (2)
N4—C58	1.338 (2)	C40—H40A	0.9900
N4—C62	1.343 (2)	C40—H40B	0.9900
C1—C2	1.390 (2)	C41—C42	1.526 (2)
C1—C6	1.400 (2)	C41—H41A	0.9900
C1—C7	1.493 (2)	C41—H41B	0.9900
C2—C3	1.393 (2)	C42—H42A	0.9800
C2—H2	0.9500	C42—H42B	0.9800
C3—C4	1.397 (2)	C42—H42C	0.9800
C3—H3	0.9500	C43—C44	1.389 (2)
C4—C5	1.399 (2)	C43—C48	1.396 (2)
C5—C6	1.381 (2)	C43—C49	1.491 (2)
C5—H5	0.9500	C44—C45	1.388 (2)
C6—H6	0.9500	C44—H44	0.9500
C8—C9	1.512 (2)	C45—C46	1.393 (2)
C8—H8A	0.9900	C45—H45	0.9500
C8—H8B	0.9900	C46—C47	1.403 (2)
C9—C10	1.524 (2)	C47—C48	1.383 (2)
C9—H9A	0.9900	C47—H47	0.9500
C9—H9B	0.9900	C48—H48	0.9500
C10—H10A	0.9800	C50—C51	1.513 (2)
C10—H10B	0.9800	C50—H50A	0.9900
C10—H10C	0.9800	C50—H50B	0.9900
C11—C12	1.388 (2)	C51—C52	1.521 (2)
C11—C16	1.399 (2)	C51—H51A	0.9900
C11—C17	1.490 (2)	C51—H51B	0.9900
C12—C13	1.393 (2)	C52—H52A	0.9800
C12—H12	0.9500	C52—H52B	0.9800
C13—C14	1.399 (2)	C52—H52C	0.9800
C13—H13	0.9500	C53—C54	1.384 (2)
C14—C15	1.399 (2)	C53—H53	0.9500
C15—C16	1.383 (2)	C54—C55	1.400 (2)
C15—H15	0.9500	C54—H54	0.9500
C16—H16	0.9500	C55—C56	1.398 (2)
C18—C19	1.514 (2)	C55—C63	1.467 (2)
C18—H18A	0.9900	C56—C57	1.380 (2)
C18—H18B	0.9900	C56—H56	0.9500
C19—C20	1.527 (2)	C57—H57	0.9500
C19—H19A	0.9900	C58—C59	1.384 (2)
C19—H19B	0.9900	C58—H58	0.9500
C20—H20A	0.9800	C59—C60	1.398 (2)
C20—H20B	0.9800	C59—H59	0.9500
C20—H20C	0.9800	C60—C61	1.399 (2)
C21—C22	1.388 (2)	C60—C64	1.464 (2)
C21—H21	0.9500	C61—C62	1.383 (2)
C22—C23	1.397 (2)	C61—H61	0.9500
C22—H22	0.9500	C62—H62	0.9500
C23—C24	1.398 (2)	C63—C64	1.328 (2)
C23—C31	1.469 (2)	C63—H63	0.9500
C24—C25	1.384 (2)	C64—H64	0.9500
			
C7—O1—H1	111 (2)	C32—C31—C23	125.44 (14)
C4—O3—C8	118.22 (12)	C32—C31—H31	117.3
C17—O4—H4	112.7 (17)	C23—C31—H31	117.3
C14—O6—C18	117.98 (12)	C31—C32—C28	125.19 (14)
C39—O7—H7	106.7 (16)	C31—C32—H32	117.4
C36—O9—C40	117.42 (12)	C28—C32—H32	117.4
C49—O10—H10D	108.7 (18)	C38—C33—C34	119.14 (14)
C46—O12—C50	117.43 (12)	C38—C33—C39	119.12 (13)
C21—N1—C25	117.43 (14)	C34—C33—C39	121.74 (14)
C26—N2—C30	117.97 (14)	C35—C34—C33	120.37 (14)
C57—N3—C53	118.04 (14)	C35—C34—H34	119.8
C58—N4—C62	118.30 (14)	C33—C34—H34	119.8
C2—C1—C6	119.07 (14)	C34—C35—C36	120.13 (15)
C2—C1—C7	121.97 (14)	C34—C35—H35	119.9
C6—C1—C7	118.96 (14)	C36—C35—H35	119.9
C1—C2—C3	121.26 (14)	O9—C36—C37	124.77 (14)
C1—C2—H2	119.4	O9—C36—C35	115.09 (14)
C3—C2—H2	119.4	C37—C36—C35	120.13 (15)
C2—C3—C4	118.99 (14)	C36—C37—C38	119.19 (14)
C2—C3—H3	120.5	C36—C37—H37	120.4
C4—C3—H3	120.5	C38—C37—H37	120.4
O3—C4—C3	124.59 (14)	C33—C38—C37	121.03 (14)
O3—C4—C5	115.24 (13)	C33—C38—H38	119.5
C3—C4—C5	120.17 (14)	C37—C38—H38	119.5
C6—C5—C4	120.07 (14)	O8—C39—O7	123.80 (15)
C6—C5—H5	120.0	O8—C39—C33	122.64 (14)
C4—C5—H5	120.0	O7—C39—C33	113.56 (13)
C5—C6—C1	120.43 (14)	O9—C40—C41	108.40 (13)
C5—C6—H6	119.8	O9—C40—H40A	110.0
C1—C6—H6	119.8	C41—C40—H40A	110.0
O2—C7—O1	123.45 (15)	O9—C40—H40B	110.0
O2—C7—C1	122.46 (14)	C41—C40—H40B	110.0
O1—C7—C1	114.09 (13)	H40A—C40—H40B	108.4
O3—C8—C9	106.97 (13)	C40—C41—C42	109.57 (14)
O3—C8—H8A	110.3	C40—C41—H41A	109.8
C9—C8—H8A	110.3	C42—C41—H41A	109.8
O3—C8—H8B	110.3	C40—C41—H41B	109.8
C9—C8—H8B	110.3	C42—C41—H41B	109.8
H8A—C8—H8B	108.6	H41A—C41—H41B	108.2
C8—C9—C10	110.65 (15)	C41—C42—H42A	109.5
C8—C9—H9A	109.5	C41—C42—H42B	109.5
C10—C9—H9A	109.5	H42A—C42—H42B	109.5
C8—C9—H9B	109.5	C41—C42—H42C	109.5
C10—C9—H9B	109.5	H42A—C42—H42C	109.5
H9A—C9—H9B	108.1	H42B—C42—H42C	109.5
C9—C10—H10A	109.5	C44—C43—C48	119.23 (14)
C9—C10—H10B	109.5	C44—C43—C49	121.69 (14)
H10A—C10—H10B	109.5	C48—C43—C49	119.08 (14)
C9—C10—H10C	109.5	C45—C44—C43	121.20 (14)
H10A—C10—H10C	109.5	C45—C44—H44	119.4
H10B—C10—H10C	109.5	C43—C44—H44	119.4
C12—C11—C16	119.11 (14)	C44—C45—C46	119.15 (14)
C12—C11—C17	120.14 (14)	C44—C45—H45	120.4
C16—C11—C17	120.74 (13)	C46—C45—H45	120.4
C11—C12—C13	121.24 (14)	O12—C46—C45	124.42 (14)
C11—C12—H12	119.4	O12—C46—C47	115.30 (14)
C13—C12—H12	119.4	C45—C46—C47	120.27 (14)
C12—C13—C14	119.08 (14)	C48—C47—C46	119.65 (14)
C12—C13—H13	120.5	C48—C47—H47	120.2
C14—C13—H13	120.5	C46—C47—H47	120.2
O6—C14—C15	115.27 (13)	C47—C48—C43	120.49 (14)
O6—C14—C13	124.68 (14)	C47—C48—H48	119.8
C15—C14—C13	120.04 (14)	C43—C48—H48	119.8
C16—C15—C14	120.05 (14)	O11—C49—O10	123.42 (15)
C16—C15—H15	120.0	O11—C49—C43	122.13 (14)
C14—C15—H15	120.0	O10—C49—C43	114.45 (13)
C15—C16—C11	120.48 (14)	O12—C50—C51	107.92 (12)
C15—C16—H16	119.8	O12—C50—H50A	110.1
C11—C16—H16	119.8	C51—C50—H50A	110.1
O5—C17—O4	123.44 (15)	O12—C50—H50B	110.1
O5—C17—C11	122.65 (14)	C51—C50—H50B	110.1
O4—C17—C11	113.90 (13)	H50A—C50—H50B	108.4
O6—C18—C19	107.83 (13)	C50—C51—C52	109.96 (13)
O6—C18—H18A	110.1	C50—C51—H51A	109.7
C19—C18—H18A	110.1	C52—C51—H51A	109.7
O6—C18—H18B	110.1	C50—C51—H51B	109.7
C19—C18—H18B	110.1	C52—C51—H51B	109.7
H18A—C18—H18B	108.5	H51A—C51—H51B	108.2
C18—C19—C20	110.60 (13)	C51—C52—H52A	109.5
C18—C19—H19A	109.5	C51—C52—H52B	109.5
C20—C19—H19A	109.5	H52A—C52—H52B	109.5
C18—C19—H19B	109.5	C51—C52—H52C	109.5
C20—C19—H19B	109.5	H52A—C52—H52C	109.5
H19A—C19—H19B	108.1	H52B—C52—H52C	109.5
C19—C20—H20A	109.5	N3—C53—C54	122.40 (14)
C19—C20—H20B	109.5	N3—C53—H53	118.8
H20A—C20—H20B	109.5	C54—C53—H53	118.8
C19—C20—H20C	109.5	C53—C54—C55	119.78 (14)
H20A—C20—H20C	109.5	C53—C54—H54	120.1
H20B—C20—H20C	109.5	C55—C54—H54	120.1
N1—C21—C22	122.88 (15)	C56—C55—C54	117.25 (14)
N1—C21—H21	118.6	C56—C55—C63	123.26 (14)
C22—C21—H21	118.6	C54—C55—C63	119.49 (14)
C21—C22—C23	119.88 (14)	C57—C56—C55	119.19 (14)
C21—C22—H22	120.1	C57—C56—H56	120.4
C23—C22—H22	120.1	C55—C56—H56	120.4
C22—C23—C24	117.02 (14)	N3—C57—C56	123.33 (14)
C22—C23—C31	119.10 (14)	N3—C57—H57	118.3
C24—C23—C31	123.88 (14)	C56—C57—H57	118.3
C25—C24—C23	119.45 (14)	N4—C58—C59	122.44 (14)
C25—C24—H24	120.3	N4—C58—H58	118.8
C23—C24—H24	120.3	C59—C58—H58	118.8
N1—C25—C24	123.32 (14)	C58—C59—C60	119.80 (14)
N1—C25—H25	118.3	C58—C59—H59	120.1
C24—C25—H25	118.3	C60—C59—H59	120.1
N2—C26—C27	122.98 (14)	C59—C60—C61	117.33 (14)
N2—C26—H26	118.5	C59—C60—C64	119.10 (14)
C27—C26—H26	118.5	C61—C60—C64	123.57 (14)
C26—C27—C28	119.58 (14)	C62—C61—C60	119.30 (14)
C26—C27—H27	120.2	C62—C61—H61	120.4
C28—C27—H27	120.2	C60—C61—H61	120.4
C29—C28—C27	116.88 (14)	N4—C62—C61	122.83 (14)
C29—C28—C32	123.92 (14)	N4—C62—H62	118.6
C27—C28—C32	119.20 (14)	C61—C62—H62	118.6
C30—C29—C28	119.85 (15)	C64—C63—C55	124.72 (14)
C30—C29—H29	120.1	C64—C63—H63	117.6
C28—C29—H29	120.1	C55—C63—H63	117.6
N2—C30—C29	122.72 (15)	C63—C64—C60	125.87 (14)
N2—C30—H30	118.6	C63—C64—H64	117.1
C29—C30—H30	118.6	C60—C64—H64	117.1
			
C6—C1—C2—C3	0.6 (2)	C38—C33—C34—C35	1.0 (2)
C7—C1—C2—C3	−179.08 (14)	C39—C33—C34—C35	−178.71 (14)
C1—C2—C3—C4	−0.7 (2)	C33—C34—C35—C36	−0.6 (2)
C8—O3—C4—C3	0.9 (2)	C40—O9—C36—C37	3.9 (2)
C8—O3—C4—C5	−179.18 (13)	C40—O9—C36—C35	−176.38 (13)
C2—C3—C4—O3	179.98 (14)	C34—C35—C36—O9	−179.49 (14)
C2—C3—C4—C5	0.1 (2)	C34—C35—C36—C37	0.2 (2)
O3—C4—C5—C6	−179.34 (13)	O9—C36—C37—C38	179.53 (14)
C3—C4—C5—C6	0.6 (2)	C35—C36—C37—C38	−0.2 (2)
C4—C5—C6—C1	−0.7 (2)	C34—C33—C38—C37	−0.9 (2)
C2—C1—C6—C5	0.1 (2)	C39—C33—C38—C37	178.79 (14)
C7—C1—C6—C5	179.75 (14)	C36—C37—C38—C33	0.5 (2)
C2—C1—C7—O2	176.28 (16)	C38—C33—C39—O8	−4.7 (2)
C6—C1—C7—O2	−3.4 (2)	C34—C33—C39—O8	174.99 (16)
C2—C1—C7—O1	−3.6 (2)	C38—C33—C39—O7	175.30 (14)
C6—C1—C7—O1	176.79 (14)	C34—C33—C39—O7	−5.0 (2)
C4—O3—C8—C9	−178.66 (13)	C36—O9—C40—C41	−178.73 (13)
O3—C8—C9—C10	−178.38 (13)	O9—C40—C41—C42	179.64 (14)
C16—C11—C12—C13	−0.3 (2)	C48—C43—C44—C45	−0.7 (2)
C17—C11—C12—C13	179.37 (14)	C49—C43—C44—C45	178.62 (14)
C11—C12—C13—C14	0.3 (2)	C43—C44—C45—C46	0.3 (2)
C18—O6—C14—C15	179.40 (13)	C50—O12—C46—C45	−5.4 (2)
C18—O6—C14—C13	−0.7 (2)	C50—O12—C46—C47	174.21 (14)
C12—C13—C14—O6	179.86 (14)	C44—C45—C46—O12	−179.82 (14)
C12—C13—C14—C15	−0.3 (2)	C44—C45—C46—C47	0.6 (2)
O6—C14—C15—C16	−179.79 (14)	O12—C46—C47—C48	179.30 (15)
C13—C14—C15—C16	0.3 (2)	C45—C46—C47—C48	−1.1 (2)
C14—C15—C16—C11	−0.4 (2)	C46—C47—C48—C43	0.7 (3)
C12—C11—C16—C15	0.3 (2)	C44—C43—C48—C47	0.3 (2)
C17—C11—C16—C15	−179.31 (14)	C49—C43—C48—C47	−179.13 (15)
C12—C11—C17—O5	−0.6 (2)	C44—C43—C49—O11	−172.16 (16)
C16—C11—C17—O5	179.02 (16)	C48—C43—C49—O11	7.2 (2)
C12—C11—C17—O4	−179.96 (14)	C44—C43—C49—O10	7.9 (2)
C16—C11—C17—O4	−0.3 (2)	C48—C43—C49—O10	−172.77 (14)
C14—O6—C18—C19	−179.70 (12)	C46—O12—C50—C51	−179.65 (13)
O6—C18—C19—C20	−179.83 (13)	O12—C50—C51—C52	179.89 (13)
C25—N1—C21—C22	0.0 (2)	C57—N3—C53—C54	0.6 (2)
N1—C21—C22—C23	1.0 (2)	N3—C53—C54—C55	−0.2 (2)
C21—C22—C23—C24	−1.0 (2)	C53—C54—C55—C56	0.1 (2)
C21—C22—C23—C31	179.48 (15)	C53—C54—C55—C63	179.36 (14)
C22—C23—C24—C25	0.1 (2)	C54—C55—C56—C57	−0.3 (2)
C31—C23—C24—C25	179.55 (15)	C63—C55—C56—C57	−179.54 (14)
C21—N1—C25—C24	−1.0 (2)	C53—N3—C57—C56	−0.8 (2)
C23—C24—C25—N1	1.0 (2)	C55—C56—C57—N3	0.7 (2)
C30—N2—C26—C27	0.2 (2)	C62—N4—C58—C59	−1.0 (2)
N2—C26—C27—C28	−1.3 (2)	N4—C58—C59—C60	0.7 (2)
C26—C27—C28—C29	1.0 (2)	C58—C59—C60—C61	0.0 (2)
C26—C27—C28—C32	−178.96 (14)	C58—C59—C60—C64	−179.28 (14)
C27—C28—C29—C30	0.3 (2)	C59—C60—C61—C62	−0.4 (2)
C32—C28—C29—C30	−179.75 (14)	C64—C60—C61—C62	178.88 (14)
C26—N2—C30—C29	1.2 (2)	C58—N4—C62—C61	0.6 (2)
C28—C29—C30—N2	−1.5 (3)	C60—C61—C62—N4	0.1 (2)
C22—C23—C31—C32	−168.18 (15)	C56—C55—C63—C64	−9.4 (2)
C24—C23—C31—C32	12.4 (2)	C54—C55—C63—C64	171.36 (15)
C23—C31—C32—C28	179.89 (15)	C55—C63—C64—C60	179.38 (14)
C29—C28—C32—C31	−13.7 (2)	C59—C60—C64—C63	−170.54 (15)
C27—C28—C32—C31	166.22 (15)	C61—C60—C64—C63	10.2 (2)

### (III) 4-n-Propoxybenzoic acid–(E)-1,2-di(pyridin-4-yl)ethene (2/1) Hydrogen-bond geometry (Å, º)

*Cg*1, *Cg*2, *Cg*3 and *Cg*4 are the centroids of the N1/C21–C25 pyridine, C1–C6 benzene, C11–C16 benzene and N4/C58–C62 pyridine rings, respectively.

**Table:**

*D*—H···*A*	*D*—H	H···*A*	*D*···*A*	*D*—H···*A*
O1—H1···N1	0.95 (4)	1.71 (4)	2.6607 (19)	175 (4)
O4—H4···N2	0.98 (3)	1.62 (3)	2.5974 (19)	179 (4)
O7—H7···N3	1.01 (2)	1.59 (2)	2.5836 (18)	170 (3)
O10—H10*D*···N4	0.97 (3)	1.63 (3)	2.5950 (18)	179 (3)
C21—H21···O2	0.95	2.50	3.193 (2)	130
C21—H21···O11	0.95	2.50	3.124 (2)	123
C26—H26···O7^i^	0.95	2.55	3.236 (2)	129
C30—H30···O8^ii^	0.95	2.47	3.155 (2)	129
C57—H57···O2^iii^	0.95	2.45	3.146 (2)	130
C58—H58···O11	0.95	2.51	3.165 (2)	126
C62—H62···O5^iv^	0.95	2.37	3.057 (2)	129
C8—H8*A*···*Cg*1^v^	0.99	2.85	3.6843 (19)	143
C8—H8*B*···*Cg*3^vi^	0.99	2.83	3.7013 (19)	147
C18—H18*A*···*Cg*2^vii^	0.99	2.80	3.5897 (19)	137
C50—H50*B*···*Cg*4^viii^	0.99	2.79	3.5721 (18)	136

Symmetry codes: (i) *x*−1, −*y*+1, *z*+1/2; (ii) *x*−1, −*y*, *z*+1/2; (iii) *x*+1, *y*, *z*; (iv) *x*, −*y*, *z*−1/2; (v) *x*, −*y*+1, *z*−1/2; (vi) *x*−1, *y*, *z*−1; (vii) *x*+1, *y*, *z*+1; (viii) *x*−1, *y*, *z*.

### (IV) 4-n-Butoxybenzoic acid–(E)-1,2-di(pyridin-4-yl)ethene (2/1) Crystal data

**Table:**

2C_11_H_14_O_3_·C_12_H_10_N_2_	*Z* = 1
*M**_r_* = 570.68	*F*(000) = 304.00
Triclinic, *P*1	*D*_x_ = 1.305 Mg m^−^^3^
Hall symbol: -P 1	Mo *K*α radiation, λ = 0.71075 Å
*a* = 7.103 (4) Å	Cell parameters from 7992 reflections
*b* = 9.060 (5) Å	θ = 3.1–30.0°
*c* = 11.627 (7) Å	µ = 0.09 mm^−^^1^
α = 82.29 (2)°	*T* = 93 K
β = 78.54 (3)°	Block, colorless
γ = 86.79 (3)°	0.49 × 0.21 × 0.10 mm
*V* = 726.3 (7) Å^3^	

### (IV) 4-n-Butoxybenzoic acid–(E)-1,2-di(pyridin-4-yl)ethene (2/1) Data collection

**Table:**

Rigaku R-AXIS RAPIDII diffractometer	2919 reflections with *I* > 2σ(*I*)
Detector resolution: 10.000 pixels mm^-1^	*R*_int_ = 0.033
ω scans	θ_max_ = 27.5°, θ_min_ = 3.1°
Absorption correction: multi-scan (ABSCOR; Higashi, 1995)	*h* = −9→9
*T*_min_ = 0.841, *T*_max_ = 0.991	*k* = −11→10
7250 measured reflections	*l* = −15→15
3311 independent reflections	

### (IV) 4-n-Butoxybenzoic acid–(E)-1,2-di(pyridin-4-yl)ethene (2/1) Refinement

**Table:**

Refinement on *F*^2^	0 restraints
Least-squares matrix: full	Hydrogen site location: mixed
*R*[*F*^2^ > 2σ(*F*^2^)] = 0.041	H atoms treated by a mixture of independent and constrained refinement
*wR*(*F*^2^) = 0.118	*w* = 1/[σ^2^(*F*_o_^2^) + (0.0831*P*)^2^ + 0.0445*P*] where *P* = (*F*_o_^2^ + 2*F*_c_^2^)/3
*S* = 1.08	(Δ/σ)_max_ = 0.001
3311 reflections	Δρ_max_ = 0.18 e Å^−^^3^
195 parameters	Δρ_min_ = −0.45 e Å^−^^3^

### (IV) 4-n-Butoxybenzoic acid–(E)-1,2-di(pyridin-4-yl)ethene (2/1) Special details

**Table:**

Geometry. All esds (except the esd in the dihedral angle between two l.s. planes) are estimated using the full covariance matrix. The cell esds are taken into account individually in the estimation of esds in distances, angles and torsion angles; correlations between esds in cell parameters are only used when they are defined by crystal symmetry. An approximate (isotropic) treatment of cell esds is used for estimating esds involving l.s. planes.
Refinement. Reflections were merged by SHELXL according to the crystal class for the calculation of statistics and refinement. _reflns_Friedel_fraction is defined as the number of unique Friedel pairs measured divided by the number that would be possible theoretically, ignoring centric projections and systematic absences.

### (IV) 4-n-Butoxybenzoic acid–(E)-1,2-di(pyridin-4-yl)ethene (2/1) Fractional atomic coordinates and isotropic or equivalent isotropic displacement parameters (Å^2^)

**Table:**

	*x*	*y*	*z*	*U*_iso_*/*U*_eq_	
O1	0.87741 (10)	−0.23737 (7)	0.37208 (5)	0.02330 (17)	
O2	0.77725 (10)	−0.20959 (7)	0.19912 (6)	0.02700 (18)	
O3	1.18789 (9)	−0.85943 (7)	0.22858 (6)	0.02097 (17)	
N1	0.73630 (11)	0.02600 (8)	0.40868 (7)	0.02009 (18)	
C1	0.94410 (12)	−0.43488 (9)	0.25797 (7)	0.01695 (19)	
C2	1.04332 (12)	−0.51022 (9)	0.34173 (7)	0.01810 (19)	
H2	1.0577	−0.4631	0.4076	0.022*	
C3	1.12077 (12)	−0.65193 (9)	0.33042 (7)	0.01837 (19)	
H3	1.1849	−0.7027	0.3892	0.022*	
C4	1.10454 (12)	−0.72040 (9)	0.23223 (8)	0.01745 (19)	
C5	1.00760 (13)	−0.64625 (9)	0.14720 (7)	0.01907 (19)	
H5	0.9966	−0.6921	0.0801	0.023*	
C6	0.92703 (12)	−0.50462 (9)	0.16124 (8)	0.01858 (19)	
H6	0.8593	−0.4549	0.1038	0.022*	
C7	0.85686 (12)	−0.28309 (9)	0.27212 (8)	0.0186 (2)	
C8	1.17439 (13)	−0.93751 (9)	0.13072 (8)	0.0196 (2)	
H8A	1.2305	−0.8782	0.0550	0.024*	
H8B	1.0382	−0.9549	0.1303	0.024*	
C9	1.28435 (12)	−1.08457 (9)	0.14586 (8)	0.0193 (2)	
H9A	1.2238	−1.1442	0.2204	0.023*	
H9B	1.4179	−1.0657	0.1516	0.023*	
C10	1.28714 (14)	−1.17236 (10)	0.04249 (8)	0.0241 (2)	
H10A	1.3452	−1.1112	−0.0319	0.029*	
H10B	1.1533	−1.1916	0.0377	0.029*	
C11	1.39915 (14)	−1.32056 (10)	0.05306 (9)	0.0280 (2)	
H11A	1.5311	−1.3027	0.0599	0.042*	
H11B	1.4014	−1.3696	−0.0174	0.042*	
H11C	1.3370	−1.3847	0.1235	0.042*	
C12	0.66351 (13)	0.11082 (10)	0.32383 (8)	0.0209 (2)	
H12	0.6610	0.0707	0.2528	0.025*	
C13	0.59161 (12)	0.25456 (10)	0.33492 (8)	0.0194 (2)	
H13	0.5429	0.3116	0.2719	0.023*	
C14	0.59107 (12)	0.31523 (9)	0.43924 (8)	0.01710 (19)	
C15	0.66576 (13)	0.22516 (9)	0.52787 (8)	0.0205 (2)	
H15	0.6674	0.2612	0.6007	0.025*	
C16	0.73711 (13)	0.08358 (10)	0.50919 (8)	0.0212 (2)	
H16	0.7889	0.0244	0.5700	0.025*	
C17	0.51374 (12)	0.46721 (9)	0.45065 (8)	0.0182 (2)	
H17	0.4804	0.5238	0.3823	0.022*	
H1	0.823 (3)	−0.133 (3)	0.3863 (19)	0.100 (7)*	

### (IV) 4-n-Butoxybenzoic acid–(E)-1,2-di(pyridin-4-yl)ethene (2/1) Atomic displacement parameters (Å^2^)

**Table:**

	*U*^11^	*U*^22^	*U*^33^	*U*^12^	*U*^13^	*U*^23^
O1	0.0321 (4)	0.0185 (3)	0.0208 (3)	0.0048 (3)	−0.0079 (3)	−0.0060 (2)
O2	0.0332 (4)	0.0217 (3)	0.0291 (4)	0.0062 (3)	−0.0145 (3)	−0.0044 (3)
O3	0.0279 (3)	0.0153 (3)	0.0225 (3)	0.0045 (2)	−0.0106 (3)	−0.0062 (2)
N1	0.0197 (4)	0.0160 (3)	0.0244 (4)	−0.0004 (3)	−0.0034 (3)	−0.0033 (3)
C1	0.0166 (4)	0.0162 (4)	0.0174 (4)	−0.0017 (3)	−0.0018 (3)	−0.0015 (3)
C2	0.0197 (4)	0.0191 (4)	0.0160 (4)	−0.0019 (3)	−0.0034 (3)	−0.0036 (3)
C3	0.0191 (4)	0.0187 (4)	0.0178 (4)	0.0004 (3)	−0.0055 (3)	−0.0016 (3)
C4	0.0176 (4)	0.0150 (4)	0.0196 (4)	−0.0010 (3)	−0.0035 (3)	−0.0015 (3)
C5	0.0236 (4)	0.0175 (4)	0.0176 (4)	−0.0019 (3)	−0.0063 (3)	−0.0038 (3)
C6	0.0208 (4)	0.0170 (4)	0.0183 (4)	−0.0009 (3)	−0.0064 (3)	0.0002 (3)
C7	0.0177 (4)	0.0179 (4)	0.0201 (4)	−0.0023 (3)	−0.0027 (3)	−0.0022 (3)
C8	0.0246 (4)	0.0173 (4)	0.0188 (4)	0.0003 (3)	−0.0074 (3)	−0.0045 (3)
C9	0.0205 (4)	0.0159 (4)	0.0230 (4)	0.0007 (3)	−0.0067 (3)	−0.0042 (3)
C10	0.0302 (5)	0.0195 (4)	0.0237 (4)	0.0015 (3)	−0.0061 (4)	−0.0066 (3)
C11	0.0269 (5)	0.0219 (4)	0.0354 (5)	0.0018 (4)	−0.0029 (4)	−0.0102 (4)
C12	0.0214 (4)	0.0211 (4)	0.0210 (4)	−0.0007 (3)	−0.0032 (3)	−0.0063 (3)
C13	0.0191 (4)	0.0200 (4)	0.0191 (4)	0.0007 (3)	−0.0051 (3)	−0.0010 (3)
C14	0.0152 (4)	0.0150 (4)	0.0210 (4)	−0.0010 (3)	−0.0035 (3)	−0.0016 (3)
C15	0.0252 (4)	0.0161 (4)	0.0218 (4)	0.0007 (3)	−0.0079 (3)	−0.0034 (3)
C16	0.0244 (4)	0.0162 (4)	0.0236 (4)	0.0016 (3)	−0.0073 (3)	−0.0015 (3)
C17	0.0186 (4)	0.0145 (4)	0.0218 (4)	0.0010 (3)	−0.0060 (3)	−0.0004 (3)

### (IV) 4-n-Butoxybenzoic acid–(E)-1,2-di(pyridin-4-yl)ethene (2/1) Geometric parameters (Å, º)

**Table:**

O1—C7	1.3218 (12)	C9—C10	1.5240 (13)
O1—H1	1.03 (2)	C9—H9A	0.9900
O2—C7	1.2167 (12)	C9—H9B	0.9900
O3—C4	1.3637 (12)	C10—C11	1.5251 (14)
O3—C8	1.4392 (11)	C10—H10A	0.9900
N1—C12	1.3363 (13)	C10—H10B	0.9900
N1—C16	1.3433 (13)	C11—H11A	0.9800
C1—C6	1.3901 (13)	C11—H11B	0.9800
C1—C2	1.3969 (14)	C11—H11C	0.9800
C1—C7	1.4923 (13)	C12—C13	1.3848 (14)
C2—C3	1.3805 (13)	C12—H12	0.9500
C2—H2	0.9500	C13—C14	1.3965 (13)
C3—C4	1.3970 (13)	C13—H13	0.9500
C3—H3	0.9500	C14—C15	1.3973 (14)
C4—C5	1.3940 (13)	C14—C17	1.4665 (13)
C5—C6	1.3919 (13)	C15—C16	1.3812 (13)
C5—H5	0.9500	C15—H15	0.9500
C6—H6	0.9500	C16—H16	0.9500
C8—C9	1.5138 (13)	C17—C17^i^	1.3375 (18)
C8—H8A	0.9900	C17—H17	0.9500
C8—H8B	0.9900		
			
C7—O1—H1	116.3 (12)	C8—C9—H9B	109.4
C4—O3—C8	118.25 (7)	C10—C9—H9B	109.4
C12—N1—C16	117.89 (8)	H9A—C9—H9B	108.0
C6—C1—C2	118.62 (8)	C9—C10—C11	113.13 (8)
C6—C1—C7	120.51 (8)	C9—C10—H10A	109.0
C2—C1—C7	120.87 (8)	C11—C10—H10A	109.0
C3—C2—C1	121.11 (8)	C9—C10—H10B	109.0
C3—C2—H2	119.4	C11—C10—H10B	109.0
C1—C2—H2	119.4	H10A—C10—H10B	107.8
C2—C3—C4	119.79 (8)	C10—C11—H11A	109.5
C2—C3—H3	120.1	C10—C11—H11B	109.5
C4—C3—H3	120.1	H11A—C11—H11B	109.5
O3—C4—C5	124.92 (8)	C10—C11—H11C	109.5
O3—C4—C3	115.24 (8)	H11A—C11—H11C	109.5
C5—C4—C3	119.84 (8)	H11B—C11—H11C	109.5
C6—C5—C4	119.59 (8)	N1—C12—C13	122.92 (8)
C6—C5—H5	120.2	N1—C12—H12	118.5
C4—C5—H5	120.2	C13—C12—H12	118.5
C1—C6—C5	121.02 (8)	C12—C13—C14	119.64 (8)
C1—C6—H6	119.5	C12—C13—H13	120.2
C5—C6—H6	119.5	C14—C13—H13	120.2
O2—C7—O1	124.00 (9)	C13—C14—C15	117.01 (8)
O2—C7—C1	123.57 (8)	C13—C14—C17	119.36 (8)
O1—C7—C1	112.43 (8)	C15—C14—C17	123.63 (9)
O3—C8—C9	107.37 (7)	C16—C15—C14	119.76 (9)
O3—C8—H8A	110.2	C16—C15—H15	120.1
C9—C8—H8A	110.2	C14—C15—H15	120.1
O3—C8—H8B	110.2	N1—C16—C15	122.78 (8)
C9—C8—H8B	110.2	N1—C16—H16	118.6
H8A—C8—H8B	108.5	C15—C16—H16	118.6
C8—C9—C10	111.29 (8)	C17^i^—C17—C14	125.41 (10)
C8—C9—H9A	109.4	C17^i^—C17—H17	117.3
C10—C9—H9A	109.4	C14—C17—H17	117.3
			
C6—C1—C2—C3	−0.99 (13)	C2—C1—C7—O1	−3.18 (12)
C7—C1—C2—C3	178.60 (7)	C4—O3—C8—C9	177.82 (7)
C1—C2—C3—C4	1.67 (13)	O3—C8—C9—C10	−177.05 (7)
C8—O3—C4—C5	−0.63 (13)	C8—C9—C10—C11	179.13 (7)
C8—O3—C4—C3	179.14 (7)	C16—N1—C12—C13	0.69 (13)
C2—C3—C4—O3	179.19 (7)	N1—C12—C13—C14	−0.95 (14)
C2—C3—C4—C5	−1.03 (13)	C12—C13—C14—C15	0.29 (13)
O3—C4—C5—C6	179.50 (7)	C12—C13—C14—C17	−179.66 (7)
C3—C4—C5—C6	−0.26 (13)	C13—C14—C15—C16	0.56 (13)
C2—C1—C6—C5	−0.33 (13)	C17—C14—C15—C16	−179.49 (8)
C7—C1—C6—C5	−179.92 (7)	C12—N1—C16—C15	0.23 (13)
C4—C5—C6—C1	0.95 (13)	C14—C15—C16—N1	−0.86 (14)
C6—C1—C7—O2	−4.16 (13)	C13—C14—C17—C17^i^	172.64 (10)
C2—C1—C7—O2	176.26 (8)	C15—C14—C17—C17^i^	−7.31 (17)
C6—C1—C7—O1	176.40 (7)		

Symmetry code: (i) −*x*+1, −*y*+1, −*z*+1.

### (IV) 4-n-Butoxybenzoic acid–(E)-1,2-di(pyridin-4-yl)ethene (2/1) Hydrogen-bond geometry (Å, º)

*Cg* is the centroid of the C1–C6 benzene ring.

**Table:**

*D*—H···*A*	*D*—H	H···*A*	*D*···*A*	*D*—H···*A*
O1—H1···N1	1.02 (3)	1.57 (3)	2.5912 (18)	179 (2)
C11—H11*C*···*Cg*^ii^	0.98	2.92	3.800 (2)	150

Symmetry code: (ii) *x*, *y*−1, *z*.

## References

[bb1] Aakeröy, C. B., Desper, J. & Urbina, J. F. (2005). *CrystEngComm*, **7**, 193–201.

[bb2] Burla, M. C., Caliandro, R., Camalli, M., Carrozzini, B., Cascarano, G. L., De Caro, L., Giacovazzo, C., Polidori, G., Siliqi, D. & Spagna, R. (2007). *J. Appl. Cryst.* **40**, 609–613.

[bb3] Farrugia, L. J. (2012). *J. Appl. Cryst.* **45**, 849–854.

[bb4] Groom, C. R., Bruno, I. J., Lightfoot, M. P. & Ward, S. C. (2016). *Acta Cryst.* B**72**, 171–179. 10.1107/S2052520616003954 PMC4822653 27048719

[bb5] Grunert, M., Howie, A., Kaeding, A. & Imrie, C. T. (1997). *J. Mater. Chem.* **7**, 211–214.

[bb6] Higashi (1995). *ABSCOR*. Rigaku Corporation, Tokyo, Japan.

[bb7] Kato, T., Fréchet, J. M. J., Wilson, P. G., Saito, T., Uryu, T., Fujishima, A., Jin, C. & Kaneuchi, F. (1993). *Chem. Mater.* **5**, 1094–1100.

[bb8] Kato, T., Wilson, P. G., Fujishima, A. & Fréchet, J. M. J. (1990). *Chem. Lett.* **19**, 2003–2006.

[bb9] Ma, Z.-C., Ma, A.-Q. & Wang, G.-P. (2006). *Acta Cryst.* E**62**, o1165–o1166.

[bb10] Mukherjee, A. & Desiraju, G. R. (2014). *Cryst. Growth Des.* **14**, 1375–1385.

[bb11] Ramon, G., Davies, K. & Nassimbeni, L. R. (2014). *CrystEngComm*, **16**, 5802–5810.

[bb12] Rigaku (2006). *RAPID-AUTO*. Rigaku Corporation, Tokyo, Japan.

[bb13] Rigaku (2010). *CrystalStructure*. Rigaku Corporation, Tokyo, Japan.

[bb15] Sheldrick, G. M. (2015). *Acta Cryst.* C**71**, 3–8.

[bb16] Spek, A. L. (2015). *Acta Cryst.* C**71**, 9–18. 10.1107/S2053229614024929 25567569

[bb17] Tabuchi, Y., Gotoh, K. & Ishida, H. (2015*a*). *Acta Cryst.* E**71**, 1340–1344. 10.1107/S2056989015019349 PMC4645034 26594506

[bb18] Tabuchi, Y., Gotoh, K. & Ishida, H. (2015*b*). *Acta Cryst.* E**71**, 1290–1295. 10.1107/S2056989015018435 PMC4645087 26594494

